# No robust evidence for an interaction between early-life adversity and protective factors on global and regional brain volumes

**DOI:** 10.1016/j.dcn.2022.101166

**Published:** 2022-10-25

**Authors:** Andrea P. Cortes Hidalgo, Henning Tiemeier, Stephen A. Metcalf, Maximilian Monninger, Andreas Meyer-Lindenberg, Pascal-M. Aggensteiner, Marian J. Bakermans‑Kranenburg, Tonya White, Tobias Banaschewski, Marinus H. van IJzendoorn, Nathalie E. Holz

**Affiliations:** a Department of Child and Adolescent Psychiatry/Psychology, Erasmus University Medical Center, Rotterdam, the Netherlands; b The Generation R Study Group, Erasmus University Medical Center, Rotterdam, the Netherlands; c Department of Social and Behavioral Sciences, Harvard T.H. Chan School of Public Health, Boston, USA; d Department of Public Health and Primary Care, University of Cambridge, Cambridge, UK; e Department of Child and Adolescent Psychiatry and Psychotherapy, Central Institute of Mental Health, Medical Faculty Mannheim, Heidelberg University, Mannheim, Germany; f Department of Psychiatry and Psychotherapy, Central Institute of Mental Health, Medical Faculty Mannheim, Heidelberg University, Mannheim, Germany; g Department of Clinical Child and Family Studies, and Amsterdam Public Health, Vrije Universiteit Amsterdam, Amsterdam, the Netherlands; h Department of Radiology and Nuclear Medicine, Erasmus University Medical Center, Rotterdam, the Netherlands; i Department of Psychology, Education and Child Studies, Erasmus University Rotterdam, Rotterdam, the Netherlands; j Department of Clinical, Educational and Health Psychology, UCL, University of London, London, UK; k Donders Institute, Radboud University, Nijmegen, the Netherlands; l Radboud University Medical Centre, Nijmegen, the Netherlands; m Institute of Medical Psychology and Medical Sociology, University Medical Center Schleswig Holstein, Kiel University, Kiel, Germany

**Keywords:** Brain morphology, Resilience, Adversity, Magnetic resonance imaging, Child

## Abstract

Childhood adversity is associated with brain morphology and poor psychological outcomes, and evidence of protective factors counteracting childhood adversity effects on neurobiology is scarce. We examined the interplay of childhood adversity with protective factors in relation to brain morphology in two independent longitudinal cohorts, the Generation R Study (N = 3008) and the Mannheim Study of Children at Risk (MARS) (N = 179). Cumulative exposure to 12 adverse events was assessed across childhood until age 9 years in Generation R and 11 years in MARS. Protective factors (temperament, cognition, self-esteem, maternal sensitivity, friendship quality) were assessed at various time-points during childhood. Global brain volumes and volumes of amygdala, hippocampus, and the anterior cingulate, medial orbitofrontal and rostral middle frontal cortices were assessed with anatomical scans at 10 years in Generation R and at 25 years in MARS. Childhood adversity was related to smaller cortical grey matter, cerebral white matter, and cerebellar volumes in children. Also, no buffering effects of protective factors on the association between adversity and the brain outcomes survived multiple testing correction. We found no robust evidence for an interaction between protective factors and childhood adversity on broad brain structural measures. Small interaction effects observed in one cohort only warrant further investigation.

## 1 Introduction

The cumulative exposure to adversities, such as parental loss and physical abuse, has been robustly related to long-lasting psychiatric problems throughout life, including behaviour, mood, anxiety, and substance disorders (Kessler et al., 2010, McLaughlin et al., 2019), accounting for about 30% of these psychopathologies in adulthood (Kessler et al., 2010). Evidence also suggests biological consequences of early-life adversities, with multiple studies showing brain morphological differences in individuals exposed to childhood adversity (McLaughlin et al., 2019, Monninger et al., 2019), and some of these findings are described in both children and adults, suggesting potential short- as well as long-term effects on brain structure (Holz et al., 2020). In general, adversity has been associated with smaller global brain volumes, suggesting a widespread effect on brain development, but also with the volume and functioning of systems involved in the response to threat and emotion (see reviews: Holz et al., 2020; Bick and Nelson, 2016, and McLaughlin et al., 2019). In particular, adversity has been related to alterations in the limbic system, which includes the amygdala, hippocampus, and the medial prefrontal cortex (including the anterior cingulate cortex (ACC)), and in two additional regions, the orbitofrontal cortex (OFC) and the dorsolateral prefrontal cortex (dlPFC), which are involved in the regulation of emotional processing and decision making (Keuper et al., 2018, Kringelbach, 2005).

Interestingly, not all individuals exposed to childhood adversity show these psychopathological and neurobiological alterations, and instead achieve healthy psychological outcomes, which could be attributed to the presence of protective or resilience factors (Brody et al., 2016, Ellis et al., 2017, Ioannidis et al., 2020, McEwen et al., 2015). However, while there is evidence for the relation between early-life adverse events and brain structure, little is known about protective factors that could counteract these effects. For example, optimism (Dolcos et al., 2016), positive coping styles (Holz et al., 2016), maternal sensitivity (Kok et al., 2015), smooth parent-child communication (Holmes et al., 2018), and having close social contacts (Taebi et al., 2020), were associated with brain morphological differences, particularly in areas that are related to adversity and that are implicated in emotion, cognition, stress regulation and affective processing, including the OFC, ACC and subcortical limbic structures (Holz et al., 2020). Some of these differences were, in turn, related to better psychosocial outcomes (Dolcos et al., 2016, Holmes et al., 2018). Further, there is some evidence for a moderating role of protective factors on the association between adversity and neurocognitive outcomes. Research is largely consistent on the adversity-buffering effect of sensitive and supportive parenting on brain outcomes (Brody et al., 2017, Holz et al., 2018, Holz et al., 2021, Morgan et al., 2014, Rakesh et al., 2021, Whittle et al., 2017). For instance, in a large cross-sectional study of children the association of neighbourhood disadvantage with altered resting-state functional connectivity was buffered by positive parenting and a favourable school environment (Rakesh, Seguin, et al., 2021). An independent intervention study showed that promoting supportive parenting in late childhood buffered the link between poverty during adolescence and smaller limbic volumes in adulthood (Brody et al., 2017). Moreover, protective effects of parental care have been suggested across biological levels, such as inflammation markers and epigenetic aging (Brody et al., 2017, Brody et al., 2016). Regarding further social factors, positive social engagement, including social bonds and friendships, has also shown poverty-counteracting effects on hippocampal volume (Ku et al., 2022). In addition, individual characteristics such as the degree of child temperamental effortful control mitigated the effect of neighbourhood disadvantage on brain structural developmental trajectories (Rakesh, Cropley, et al., 2021). Similarly, three temperament dimensions – high effortful control, low surgency, and low negative affectivity – attenuated the link between socioeconomic disadvantage and poor child academic outcomes (Wang et al., 2017). Further, child characteristics such as self-concept and self-esteem have also been identified as resource factors related to resilience with regard to mental health outcomes (Miller-Lewis et al., 2013) and hippocampal volumes (Wang et al., 2016) in children and adults, respectively, exposed to adversity. Lastly, a recent systematic review on childhood resilience identified cognitive abilities as a resilience-promoting factor in children exposed to adversity (Gartland et al., 2019). Overall, previous research supports a role of multiple protective factors, including parental care, child temperament, self-esteem, cognitive abilities and social relationships. However, most evidence is based on *functional* brain and psychological outcomes, and the few studies on brain structure mostly focused on specific adverse events like poverty, and did not assess the occurrence of adversity in early childhood.

Identifying the moderating role of protective factors would help us understand which factors potentially represent intervention targets for further evaluation in observational and intervention studies (Brody et al., 2016, Gartland et al., 2019, Holz et al., 2018). Importantly, such research needs longitudinal developmental approaches, to examine how adversity and protective factors interact on brain outcomes at different stages of life (Brody et al., 2016). Ultimately, the goal of this research is to understand the counteracting effect of protective factors that can help reduce the biological, psychological and cognitive negative sequelae of the adversity exposure. Thus, we addressed our research question using two independent longitudinal cohorts, Generation R (N = 3008 in the analyses) and the Mannheim Study of Children at Risk (MARS, N = 179 in the analyses) with the aim of exploring whether similar findings would be observed across different developmental stages. While the first cohort is ideal for studying brain outcomes shortly after the adversity exposure, i.e. in late childhood, the second cohort may provide evidence on longer term associations, as the brain structures were assessed in adulthood. Also, whereas Generation R is a diverse population-based cohort, MARS is oversampled for high-risk participants, complementing the first study with a sample more exposed to adversities. Both birth cohorts provide a rich set of data on adversities and protective factors assessed from early childhood onwards, allowing us to achieve good harmonisation across cohorts. The use of both samples provided an opportunity to examine the developmental generalisability of our results.

We focused on the occurrence of *cumulative* adversity based on previous evidence (Burt et al., 2016, Miller-Lewis et al., 2013) and on data availability in both cohorts, and on the presence of protective factors during childhood, given the relevance of this developmental period. The brain goes through rapid and substantial changes during foetal life and childhood, including synaptogenesis, dendritic growth, and myelination (Lyall et al., 2015). In this dynamic maturation process, neuroplasticity is heightened and environmental influences may have long-lasting effects (White, 2019). Following evidence for the protective factor model (Holz et al., 2020), we analysed whether adversity interacts with various protective factors during childhood to shape brain morphology. Additionally, considering that the moderating effect of protective factors on adversity effects may not be the only pathway to resilience, we also explored whether the presence of protective factors per se was associated with differences in brain structure, as an alternative mechanism in which protective factors may enhance the capacity to adapt after the exposure to adversity (Ioannidis et al., 2020). Based on previous literature, we focused on the limbic system, the medial OFC and the rostral middle frontal cortex (RMF), since their role in the regulation of stress and affective processing may be particularly relevant for resilient outcomes after early-life adversity (Holz et al., 2020, McLaughlin et al., 2019). Considering that some commonalities have been suggested between brain correlates of resilience in young people and adults (Eaton et al., 2022), we hypothesised that in both Generation R and MARS, smaller volumes of the regions of interest would be observed in participants with childhood adversities and that these associations would be buffered or counteracted by a priori defined protective factors.

## 2 Methods

### 2.1 Participants

**Fig. 1:**
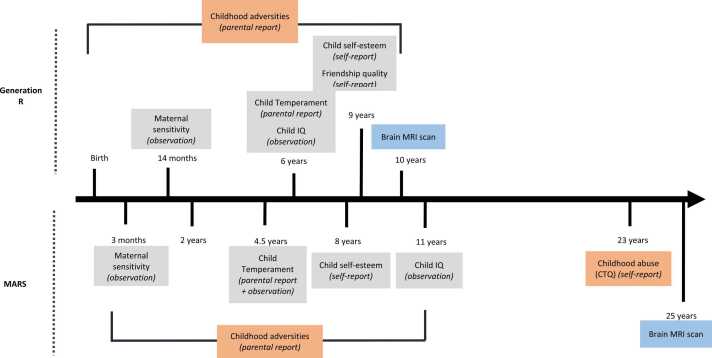
Timeline of data collection for the main variables of interest in Generation R and MARS. Note. In grey: protective factors. In blue: MRI scans. In orange: adversity measures. All measures were prospectively collected except for: childhood abuse (CTQ) in MARS, and some childhood adversity questions in Generation R (for more detail see Table 2).

We used data from two ongoing prospective birth cohort studies, the Generation R Study and MARS (Fig. 1). Adversity was assessed in both studies using multiple measures collected during childhood (from birth to age 9 years in Generation R and 11 years in MARS – except measures of childhood abuse reported by participants in MARS at age 23 years), brain images were collected at age 10 years in Generation R and at 25 years in MARS. Protective factors were assessed at different time points during childhood in both cohorts.

#### 2.1.1 Generation R

The Generation R Study is a population-based cohort study that follows the development of children in Rotterdam, the Netherlands (Kooijman et al., 2016). Pregnant women with an expected delivery date between April 2002 and January 2006 were invited to participate, and 9778 women were enroled in the study (response rate at birth: 61 %). The study was approved by the Medical Ethical Committee of the Erasmus Medical Center, and all parents gave written informed consent. Overall, 6882 children had information available on at least 50 % of the childhood adversity measures. Among this sample, structural brain magnetic resonance imaging (MRI) scans were obtained in 3925 nine-to-eleven-year-old children (White et al., 2018). We excluded children with poor image quality data (N = 763), and one sibling selected at random from each sibling pair to avoid bias due to paired data (N = 154). In total, 3008 children were included in the analyses (Fig. S1).

#### 2.1.2 MARS

This birth cohort follows the development of participants since early life to study the long-term outcomes of early risk factors (Laucht et al., 2000). Inclusion of infants was based on a two-factorial design (factor one as the presence of obstetric complications, and factor two as psychosocial adversity) to oversample infants exposed to early psychosocial and biological risk factors. Only firstborn children with predominantly European descent (> 99 %) and German-speaking parents were included. In total, 384 infants born between 1986 and 1988 were recruited from two obstetric and six children’s hospitals in the Rhine-Neckar Region of Germany. Among these participants, 18 (4.7 %) were excluded because of severe disabilities, and 57 (14.8 %) were dropouts. From the 309 participants included in the 25-year assessment, structural brain MRI data were collected in a subsample of right-handed participants with no current psychopathology. In total, 179 participants were included in our study sample (Fig. S2; Supplement). MARS was approved by the Ethics Committee of the University of Heidelberg, and all participants gave written informed consent.

### 2.2 Measures

#### 2.2.1 Childhood adversity

Given evidence on protective factors’ effects against *cumulative* adversity, with adversity being defined for example as family adversity (including events like socioeconomic problems, parental separation, early parenthood and parental psychological problems) (Miller-Lewis et al., 2013), and based on questionnaires of negative life events (Burt et al., 2016), we assessed cumulative adversity, operationalized based on previous evidence and on data availability in both cohorts. In Generation R and MARS, a sum score of childhood adversity was constructed based on the occurrence of 12 adverse events during childhood that were similar across both cohorts: early parenthood, one-parent family at child birth, unwanted pregnancy, parental psychopathology, poverty, parent’s death, family relationship problems, parental divorcee/separation, unemployment, physical abuse, psychological abuse, and sexual abuse. Data on these events were collected primarily during childhood in both cohorts (Table S1; Supplement) and were, for the most part, prospectively reported by parents or caregivers. All adversities were dichotomised based on the occurrence of the event (yes/no), using thresholds established by the literature when needed (e.g., psychopathology symptoms (De Beurs, 2004)). When repeated measures of adverse events were available (e.g., from data collected at different time points), we combined the measures into one variable based on whether the event had ever occurred in childhood. This was done because repeated measures were not available for all events, precluding the counting of occurrences.

#### 2.2.2 Childhood protective factors

Protective factors were selected based on previous research (Ellis et al., 2017, Holz et al., 2020, Wang et al., 2016) and were measured during childhood in both cohorts. We included child temperament, child intelligence quotient (IQ), child self-esteem, maternal sensitivity (only available for a subsample in Generation R), and friendship quality (only included in Generation R since no comparable measure was available in MARS) (Table S2; Supplement).

Child temperament was reported by the main caregiver at child age 6 years in Generation R, based on the Very Short Form of the Children’s Behaviour Questionnaire (Putnam and Rothbart, 2006) (dimensions: negative affectivity (reversed in our analyses to facilitate interpretation), surgency/extraversion, and effortful control (Ghassabian et al., 2014)). In MARS, child temperament was based on a standardised parent interview and observations of the child in familiar and unfamiliar settings at age 4.5 years, using rating scales and an interview adapted from Thomas et al. (1968) (Factors extracted: easy-difficult trait and self-control).

Child IQ was assessed with a non-verbal cognition test in both cohorts, at 6 years in Generation R children using the Snijders-Oomen Nonverbal Intelligence Test (SON-R 2.5-7) (Tellegen et al., 1998), and at 11 years in MARS, with the Culture Fair Intelligence Test (CFT-20) (Cattell, 1960).

Child self-esteem was reported by children at age 9 years in Generation R and at age 8 years in MARS. In Generation R, global self-esteem was assessed based on the Dutch version of Harter’s Self-Perception Profile for Children (Veerman et al., 1997), with an adapted question format based on Wichstraum (1995). In MARS, global child self-concept (referred to as self-esteem) was assessed using the German version of the Perceived Competence Scales (Asendorpf and Van Aken, 1993, Harter and Pike, 1984).

Maternal sensitivity was observed in both cohorts. In Generation R, maternal sensitivity was examined in a subsample of children with Dutch national origin (N = 383 in these analyses) during the 14-month laboratory visit (Ainsworth et al., 1974). In MARS, the interaction between the mother and the 3-month-old infant was coded using the Mannheim Rating System for Mother-Infant Interaction (Esser et al., 1989). We included adequate maternal stimulation as a measure of maternal sensitivity (Holz et al., 2018, Holz et al., 2021) and infant responsiveness was added as a covariate in these analyses to assess maternal behaviour independent of the degree of child responsiveness (Holz et al., 2018).

Friendship quality was assessed at child age 9 years in Generation R. Children rated the quality of their best friendship based on an adapted version of the Friendship Quality Questionnaire (FQQ) (Parker and Asher, 1993).

### 2.3 Brain morphology

#### 2.3.1 Generation R

At 9‐to‐11 years of age, children underwent a neuroimaging scanning session, with a 3-Tesla MRI scanner (MR750w, General Electric, Milwaukee, WI, USA) using an 8-channel receive-only head coil (White et al., 2018). T_1_-weighted structural images were obtained with a coronal inversion recovery fast spoiled gradient recalled sequence (IR-FSPGR BRAVO) (ARC acceleration factor = 2, Repetition time = 8.77 ms, Echo time = 3.4 ms, Inversion time = 600 ms, Flip angle = 10°, Field of view = 220 × 220, Acquisition matrix = 220 × 220, Slice thickness = 1 mm, Number of slices = 230).

#### 2.3.2 MARS

At 25 years of age, participants underwent the neuroimaging data collection, with a 3-Tesla MRI scanner (Magnetom TRIO, Siemens, Erlangen, Germany) using a 12-channel head coil. The 1 × 1 × 1 mm^3^ T_1_-weighted MRI scans were acquired with the following parameters: Number of slices = 192, Matrix = 256 × 256, Repetition time = 2300 ms, Echo time = 3.03 ms, 50 % distance factor, Field of view = 256 × 256 × 192 mm^3^, Flip angle = 9° (Holz et al., 2015, Monninger et al., 2019).

#### 2.3.3 Anatomical data analysis – Generation R and MARS

Neuroimaging data were processed using the FreeSurfer analysis suite (v.6.0) (Fischl, 2012). Briefly, cortical reconstruction (removal of non-brain tissue, correction of voxel intensities, voxels segmentation into white and grey matter and cerebral spinal fluid, and generation of surface-based models of white and grey matter) and volumetric segmentation were performed. Global and regional brain volume metrics were extracted and cortical vertices were automatically labelled based on the Desikan-Killiany atlas (Desikan et al., 2006).

#### 2.3.4 Regions of interest (ROIs)

Based on previous literature (Holz et al., 2020), we examined the cortical grey matter, cerebral white matter, cerebellum, amygdala, hippocampus, left and right ACC, left and right medial OFC, and left and right RMF volumes (cortical regions based on the Desikan-Killiany atlas (Desikan et al., 2006)). The ACC measure was constructed as the sum of the rostral and caudal ACC. The RMF was included as the most likely representation of the dorsolateral prefrontal cortex (Kikinis et al., 2010, Levitt et al., 2017). Left and right cortical regions were examined separately given evidence of cortical structural asymmetry (Kong et al., 2018). As in previous studies (Gehred et al., 2021, Luby et al., 2019), we averaged amygdala and hippocampal volumes across hemispheres, to reduce the number of tests in our main analyses and since we had no a priori hypothesis for laterality-specific effects. Left and right amygdala and hippocampus volumes, and the surface area and thickness of the cortical regions were studied using exploratory analyses. Cortical surface area and cortical thickness were examined considering that both undergo changes in childhood and adolescence and have distinct patterns of development (Fuhrmann et al., 2022, Lyall et al., 2015).

### 2.4 Covariates

Covariates were selected based on previous literature (Luby et al., 2019, Monninger et al., 2019, Pulli et al., 2019). Covariates included sex, total intracranial volume (included in analyses of the limbic and cortical regions), prenatal smoking (Pulli et al., 2019), maternal national origin (only in Generation R), age at MRI scan (only in Generation R, given only very small age-related effects in early adulthood (Ziegler et al., 2012)), and a measure of obstetric risk including low birth weight (Laucht et al., 2000). Additionally, child responsiveness was adjusted for in analyses with maternal sensitivity in MARS. Generation R analyses were controlled for national origin to adjust for brain structural differences (Kang et al., 2020) and social and cultural determinants that can lead to variations in childhood adversity experience. This is particularly relevant for this cohort because of its multi-ethnic composition, including a large percentage of participants from different national origin groups (Kooijman et al., 2016).

### 2.5 Statistical analyses

All analyses were performed with R statistical software (v.4.1.0) (R Core Team, 2020). Phi correlations were calculated between all adversity variables, and Spearman correlations were calculated to describe the associations across adversity and the protective factors. Multiple linear regression analyses adjusted for covariates were performed to examine the main effects of childhood adversity and the additive interactions between adversity and protective factors on the brain outcomes. The interaction effects were assessed by including a multiplicative term between cumulative adversity and the protective factor in separate models for each protective factor, implying that sample sizes could vary across analyses (e.g. analyses including maternal sensitivity performed in N = 383. All sample sizes are noted in tables’ footnotes).

We also explored the association of each protective factor with the volume of all ROIs in models adjusted for sex, total intracranial volume (in subcortical and prefrontal regions), maternal national origin (only in Generation R) and age at the MRI scan (only in Generation R), to examine whether protective factors had a direct association with brain structural outcomes.

In sensitivity analyses, we addressed additional measures of our cortical ROIs, the potential moderation by hemisphere laterality and national origin, and whether our results would be robust to different operationalizations of adversity. Specifically, we analysed the interaction of adversity with the protective factors for: 1) the surface area and cortical thickness of the cortical ROIs; 2) the left and right amygdala and hippocampus; and 3) the cortical grey matter, cerebral white matter, cerebellar, amygdala, hippocampal, ACC, medial OFC and RMF volumes only in Generation R children with mothers of European descent. The analyses conducted only in individuals of European descent were performed given growing evidence for differences in the vulnerability to the effect of adversity across national origins/ethnic groups (Roxburgh and MacArthur, 2014, Stinson et al., 2021, Youssef et al., 2017). These sensitivity analyses were not performed in the multiple minority groups as these were too small and diverse. Also, due to the correlative nature of the adverse events examined, we performed a principal component analysis (with VARIMAX rotation and phi correlations) on adverse events in Generation R and MARS to explore how these clustered and whether protective factors interacted differently with the adversity components on the ROIs (see also Supplement).

We corrected for multiple testing using the false discovery rate (FDR) (Benjamini and Hochberg, 1995) in the main analyses, separately for 1) the association between adversity and the brain outcomes (11 tests per cohort), 2) the association between protective factors and the brain outcomes (in Generation R, 7 protective factors and 11 outcomes: 77 tests, in MARS: 5 protective factors and 11 outcomes: 55 tests), and 3) in the interaction analyses between adversity and protective factors on the brain outcomes (In Generation R, 7 protective factors and 11 outcomes: 77 tests; in MARS, 5 protective factors and 11 outcomes: 55 tests). The sensitivity analyses were not corrected for multiple testing as these were exploratory (see Supplement).

All effect estimates were standardised. Analyses in MARS were performed in participants with complete data (due to few missing values). In Generation R, missing values for covariates, childhood adversity, and protective factors (maximum missingness: paternal psychopathology at child age 3 years: 42%, and maternal psychopathology at child age 6 months: 37 %) were imputed using the Multivariate Imputation by Chained Equations package (v.3.13.0) (van Buuren and Groothuis-Oudshoorn, 2011), pooling results across 40 imputed datasets. The missingness in the two psychopathology measures in Generation R is largely explained by study design. During child ages 0–4 years, data collection only included participants in northern Rotterdam due to logistical constraints. From child age 6 years onwards, all children from the initial catchment area of Rotterdam were invited to participate in follow-up assessments (Kooijman et al., 2016). Maternal sensitivity was not imputed, as it was assessed in a subsample of Generation R and values were missing for 87.3 % of the children. Non-response analyses are described in detail in the Supplement.

## 3 Results

**Fig. 2:**
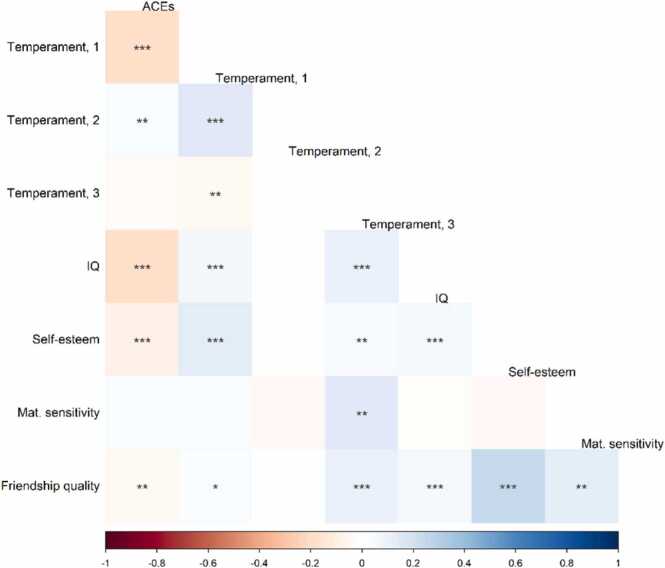
Spearman correlations between cumulative adversity and the protective factors in Generation R. Note. The colour grading shows the correlation strength. Performed in the first imputed dataset. N for all = 3008, except for correlations with maternal sensitivity (N = 383). * p < 0.05, ** p < 0.01, *** p < 0.001. ACEs = cumulative adversity; Temperament, 1 = Temperament – Negative affectivity, reversed; Temperament, 2 = Temperament – Surgency; Temperament, 3 = Temperament – Effortful control.

**Fig. 3:**
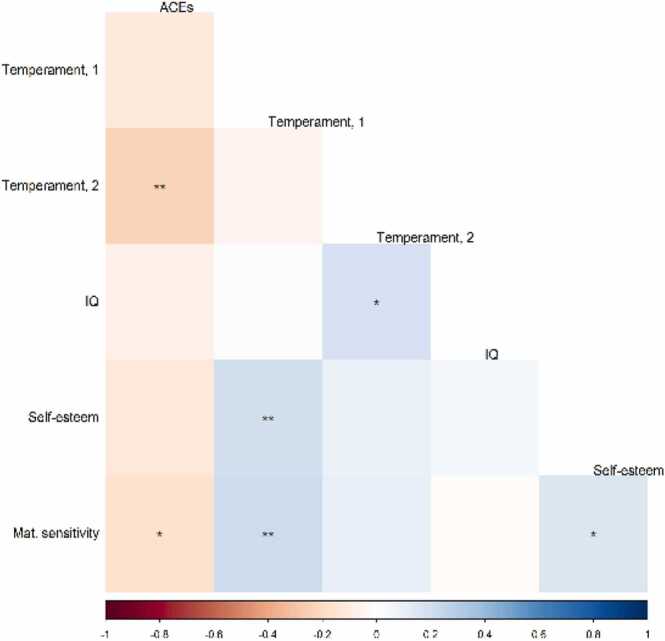
Spearman correlations between cumulative adversity and the protective factors in MARS. Note. The colour grading shows the correlation strength. N for all = 179, except for correlations with maternal sensitivity (N = 173). * p < 0.05, ** p < 0.01, *** p < 0.001. ACEs = cumulative adversity; Temperament, 1 = Temperament – easy/difficult trait; Temperament, 2 = Temperament – self-control.

The samples’ characteristics are described in Table 1. In total, 70 % of participants in Generation R, and 91 % in MARS, were exposed to at least one adversity. In both cohorts, the most common adversities were parental psychopathology (Generation R: 32.8 %, MARS: 49.2 %) and unemployment of both parents (Generation R: 31.6 %, MARS: 70.4%) (see details on protective factors and adversity in Table 2). All correlations between cumulative adversity and the protective factors were below r = 0.30 (strongest correlation observed for self-esteem and friendship quality (r = 0.25) in Generation R, and for adversity and temperament – self-control (r = − 0.23) in MARS) (Fig. 2, Fig. 3) and correlations between adverse events ranged up to 0.45 (Tables S3 and S4).

**Table 1:** Baseline characteristics of the samples from the Generation R Study and MARS.

	Generation R	MARS
***Sample characteristics***		
Sex, N (%) female	1516 (50.4)	105 (58.7)
Age at the MRI scan (years), mean (SD)	10.1 (0.6)	25.0 (0.60)
Childhood adverse events, N (%)		
0	889 (29.6)	17 (9.5)
1	803 (26.7)	36 (20.1)
2	520 (17.3)	42 (23.5)
3	361 (12)	32 (17.9)
4 or more	435 (14.4)	52 (29.0)
Maternal national origin, N (%)		
European descent	1980 (65.8)	–
Others	1028 (34.2)	–
Maternal smoking, smoking during pregnancy, N (%)	679 (22.6)	40 (22.3)

Note. Characteristics of the study sample (pooled imputed data in Generation R).

**Table 2:** Characteristics of protective factors and childhood adversity in the Generation R Study and the MARS.

**Generation R Study**							**MARS**					
**Event/Factor**		**Assessment**	**Age at assessment**	**Reporter**	**Exposed**		**Event/Factor**	**Assessment**	**Age at assessment**	**Reporter**	**Exposed**	
					**N**	%*					**N**	%*
**Adverse events**												
**1**	Early parenthood	Quest.	pregnancy	Mother	79	2.6	Early parenthood	Interv.	3 months	parents	47	26.3
**2**	One-parent family at child birth	Quest.	pregnancy	Mother	348	11.6	One-parent family at child birth	Interv.	3 months	parents	17	9.5
**3**	Unwanted pregnancy	Quest.	pregnancy	Mother	39	1.3	Unwanted pregnancy	Interv.	3 months	parents	24	13.4
**4**	Parental (maternal or paternal) psychopathology	Quest.	2 months, 6 months, 3 and 9 years	Mother and partner	987	32.8	Parental (maternal or paternal) psychopathology	Interv.	3 months, 2, 4.5, 8 and 11 years	parents	88	49.2
**5**	Poverty	Quest.	pregnancy	Mother	559	18.6	Poverty	Interv.	3 months	mother	35	19.6
**6**	Death of parent	Interv.	9 years	Careg.	28	0.9	Death of parent	2 and 4.5 years: Interv. 8 and 11 years: Quest.	2, 4.5, 8 and 11 years	parents	3	1.7
**7**	Family relationship problems	Quest.	3, 5, and 9 years	Careg./mother/partner	667	22.2	Family relationship problems	Interv.	3 months, 2, 4.5, 8 and 11 years	parents	71	39.7
**8**	Divorce/separation by age 9 years	Quest.	3, 5 and 9 years	Mother	662	22.0	Divorce/separation by age 11 years	2 and 4.5 years: Interv. 8 and 11 years: Quest.	2, 4.5, 8 and 11 years	parents	44	24.6
**9**	Unemployment	3 years: Quest. 9 years: Inter.	3 and 9 years	Careg.	952	31.6	Unemployment	2 and 4.5 years: Interv. 8 and 11 years: Quest.	2, 4.5, 8 and 11 years	parents	126	70.4
**10**	Physical abuse to child	Interv.	9 years	Careg.	210	7.0	Physical abuse to child	Quest.	23 years	participant**	5	2.8
**11**	Psychological abuse to child	Interv.	9 years	Careg.	351	11.7	Psychological abuse to child	Quest.	23 years	participant**	13	7.3
**12**	Sexual abuse	Interv.	9 years	Careg.	134	4.5	Sexual abuse	Quest.	23 years	participant**	3	1.7
	***Any category reported***				2118	70.4	***Any category reported***				162	90.5
		**Assessment**	**Age at assessment**	**Reporter**	**mean (SD)**			**Assessment**	**Age at assessment**	**Reporter**	**mean (SD)**	
**Protective factors**												
**1**	**Temperament**	Quest.	6 years	Careg.			**Temperament**	Observ. + Interv.	4.5 years	Interv.: parents		
	Temperament - Negative affectivity, reversed				-3.70 (0.83)		Temperament - easy/difficult trait				0.05 (0.96)	
	Temperament - Surgency				4.42 (0.79)		Temperament - self-control				0.14 (0.92)	
	Temperament - Effortful control				5.29 (0.68)							
**2**	**Child non-verbal IQ**	Observ.	6 years	–	102.91 (14.90)		**Child non-verbal IQ**	Observ.	11 years	–	105.68 (11.22)	
**3**	**Child self-esteem**	Quest.	9 years	Child	45.74 (4.26)		**Child self-esteem**	Quest.	8 years	Child	57.64 (6.36)	
**4**	Maternal sensitivity*	Observ.	14 months	–	0.02 (0.81)		Maternal stimulation (sensitivity)***	Observ.	3 months	–	-0.06 (2.60)	
	***Additional cohort-specific measures***											
**5**	**Friendship quality**	Quest.	9 years	Child	24.11 (3.25)		–	–	–	–	–	

Abbreviations: Quest: Questionnaire, Observ: Observation, Careg.: Main caregiver, Interv: Interview, ** Participant: self-report. When repeated measures for an adverse event were available, these were combined based on whether the event had ever occurred in childhood. Characteristics described in imputed dataset of Generation R Study.

In Generation R, N = 3008. *Maternal sensitivity in Generation R available for N = 383.

In MARS, N = 179. ***Maternal sensitivity in MARS available for N = 173.

### 3.1 Childhood adversity and brain volumes

In children, exposure to adversity was associated with smaller cortical grey matter (β = − 0.09, 95 % confidence interval (CI) − 0.13 to − 0.06, p_*uncorr*_ < 0.001, p_*corr*_ < 0.001), cerebral white matter (β = − 0.07, CI − 0.11 to − 0.04, p_*uncorr*_ < 0.001, p_*corr*_ < 0.001), and cerebellar (β = − 0.08, CI − 0.12 to − 0.05, p_*uncorr*_ < 0.001, p_*corr*_ < 0.001) volumes. In adults, the association of adversity with the cerebellar volume showed a similar effect size (β = − 0.11, CI − 0.24 to 0.01), but did not reach statistical significance (p_*uncorr*_ = 0.08). In children, adversity was also related to larger left medial OFC volume (β = 0.04, CI 0.01 to 0.08, p_*uncorr*_ = 0.01, p_*corr*_ = 0.03). No other associations were observed for the global and regional, subcortical (amygdala and hippocampus), or cortical (left and right ACC, medial OFC and RMF) volumes that survived multiple testing correction (Table 3).

**Table 3:** Associations between cumulative childhood adversity and brain outcomes.

	Generation R Study		MARS	
	β (95 %CI)	p-value	β (95 %CI)	p-value
**Brain outcomes**				
*Global and regional brain outcomes*				
Cortical grey matter volume	-0.09 (− 0.13; − 0.06)	< 0.001*	0.00 (− 0.12; 0.12)	0.96
Cerebral white matter volume	-0.07 (− 0.11; − 0.04)	< 0.001*	-0.01 (− 0.14; 0.12)	0.88
Cerebellar volume	-0.08 (− 0.12; − 0.05)	< 0.001*	-0.11 (− 0.24; 0.01)	0.08
*Subcortical outcomes*				
Amygdala	-0.01 (− 0.04; 0.02)	0.55	0.06 (− 0.04; 0.16)	0.23
Hippocampus	0.00 (− 0.03; 0.03)	0.85	0.06 (− 0.06; 0.17)	0.33
*Cortical regions*				
Left ACC volume	0.02 (− 0.01; 0.06)	0.19	-0.01 (− 0.13; 0.12)	0.93
Right ACC volume	0.00 (− 0.04; 0.03)	0.85	0.02 (− 0.11; 0.16)	0.74
Left medial OFC volume	0.04 (0.01; 0.08)	0.01*	0.00 (− 0.12; 0.11)	0.95
Right medial OFC volume	0.00 (− 0.03; 0.03)	0.84	0.01 (− 0.11; 0.12)	0.93
Left rostral middle frontal volume	0.02 (− 0.01; 0.05)	0.14	0.12 (0.02; 0.23)	0.02
Right rostral middle frontal volume	-0.02 (− 0.05; 0.01)	0.22	0.14 (0.04; 0.23)	0.01

Note. Model adjusted for sex, total intracranial volume (only in subcortical and cortical regions), prenatal smoking, maternal national origin (only in Generation R), age at the MRI scan (only in Generation R), and obstetric risk.

Adversity and brain outcomes were standardised. Amygdala and hippocampus volumes are the mean volumes across left and right hemisphere. Abbreviations: ACC: Anterior cingulate cortex, OFC: Orbitofrontal cortex

Generation R N = 3008

MARS N = 179

* p-values that survived adjustment for multiple testing (including all regions of interest, method: FDR).

### 3.2 Protective factors and brain volumes

In children, there were several associations between protective factors and volumetric differences in the global measures and the amygdala. For example, child IQ was related to larger global brain volumes (e.g. cortical grey matter volume: β = 0.13, CI 0.10 to 0.17, p_*uncorr*_ < 0.001, p_*corr*_ < 0.001), and a higher self-esteem was related to smaller amygdala in children (β = − 0.04, CI −0.07 to −0.01, p_*uncorr*_ = 0.004, p_*corr*_ = 0.04). In adults, no findings survived correction for multiple testing, although similar associations in uncorrected analyses were observed for the global brain (e.g. child IQ and cortical grey matter volume: β = 0.12, CI 0.01 to 0.23, p_*uncorr*_ = 0.03, p_*corr*_ = 0.33) and limbic volumes (self-esteem and smaller hippocampal volume: β = − 0.12, CI − 0.23 to − 0.01, p_*uncorr*_ = 0.03, p_*corr*_ = 0.33) (Table S5, Fig. S3).

### 3.3 Interaction between childhood adversity and protective factors on the brain volumes

No results survived adjustment for multiple testing, nor were they consistently observed across cohorts (Table 4).

**Table 4:** Interaction between protective factors and cumulative adversity in relation to brain outcomes.

		*Global and regional brain outcomes*													*Subcortical outcomes*							
		Cortical grey matter volume				Cerebral white matter volume				Cerebellar volume					Amygdala				Hippocampus			
		**β (95 %CI)**		**p-value**		**β (95 %CI)**			**p-value**	**β (95 %CI)**			**p-value**		**β (95 %CI)**		**p-value**		**β (95 %CI)**		**p-value**	
***Generation R Study***																						
**Temperament - Negative affectivity, reversed**		-0.01 (-0.05; 0.02)		0.48		0.00 (-0.04; 0.04)			1.00	0.01 (-0.03; 0.05)			0.61		0.00 (-0.03; 0.03)		0.92		-0.01 (-0.04; 0.02)		0.44	
**Temperament - Surgency**		0.01 (-0.02; 0.05)		0.42		0.00 (-0.04; 0.04)			0.99	0.02 (-0.02; 0.06)			0.33		0.01 (-0.02; 0.05)		0.46		-0.01 (-0.04; 0.02)		0.58	
**Temperament - Effortful control**		-0.02 (-0.05; 0.01)		0.22		-0.01 (-0.05; 0.03)			0.60	0.01 (-0.03; 0.04)			0.77		0.00 (-0.03; 0.04)		0.80		0.02 (-0.01; 0.05)		0.22	
**Child non-verbal IQ**		-0.02 (-0.06; 0.01)		0.20		-0.02 (-0.05; 0.02)			0.31	-0.01 (-0.04; 0.03)			0.73		-0.01 (-0.05; 0.02)		0.38		-0.02 (-0.05; 0.02)		0.32	
**Child self-esteem**		0.01 (-0.02; 0.04)		0.62		0.01 (-0.02; 0.04)			0.44	0.00 (-0.03; 0.04)			0.79		0.00 (-0.03; 0.03)		0.97		0.01 (-0.02; 0.04)		0.66	
Maternal sensitivity*		0.05 (-0.04; 0.13)		0.28		0.00 (-0.09; 0.08)			0.97	-0.02 (-0.10; 0.07)			0.68		0.05 (-0.02; 0.12)		0.19		0.00 (-0.07; 0.08)		0.91	
**Friendship quality**		0.02 (-0.01; 0.06)		0.22		0.03 (0.00; 0.07)			0.08	0.04 (0.00; 0.07)			0.05		-0.02 (-0.05; 0.01)		0.15		-0.01 (-0.05; 0.02)		0.37	

***MARS***																						
**Temperament**																						
Temperament – easy/difficult trait		-0.01 (-0.14; 0.11)		0.81		0.01 (-0.12; 0.14)			0.84	0.01 (-0.12; 0.14)			0.86		-0.04 (-0.14; 0.06)		0.44		-0.04 (-0.15; 0.07)		0.49	
Temperament – self-control		0.03 (-0.07; 0.13)		0.56		0.02 (-0.08; 0.13)			0.66	0.04 (-0.07; 0.14)			0.49		0.00 (-0.08; 0.08)		0.98		-0.01 (-0.10; 0.08)		0.84	
**Child non-verbal IQ**		0.01 (-0.10; 0.12)		0.89		0.04 (-0.08; 0.16)			0.47	-0.03 (-0.15; 0.09)			0.61		-0.08 (-0.18; 0.01)		0.07		-0.01 (-0.11; 0.10)		0.91	
**Child self-esteem**		-0.03 (-0.16; 0.10)		0.65		0.02 (-0.12; 0.16)			0.78	0.08 (-0.06; 0.22)			0.26		0.10 (-0.01; 0.21)		0.06		0.09 (-0.03; 0.21)		0.16	
Maternal stimulation (sensitivity)**		0.02 (-0.07; 0.12)		0.65		0.05 (-0.05; 0.15)			0.33	-0.01 (-0.11; 0.09)			0.90		0.01 (-0.07; 0.09)		0.77		0.03 (-0.06; 0.12)		0.48	
						

	Left ACC				Right ACC			Left medial OFC				Right medial OFC				Left rostral middle frontal cortex				Right rostral middle frontal cortex		
	**β (95 %CI)**		**p-value**		**β (95 %CI)**		**p-value**	**β (95 %CI)**			**p-value**	**β (95 %CI)**		**p-value**		**β (95 %CI)**		**p-value**		**β (95 %CI)**		**p-value**
***Generation R Study***																						
**Temperament - Negative affectivity, reversed**	0.01 (-0.03; 0.04)		0.76		-0.02 (-0.06; 0.02)		0.27	-0.02 (-0.05; 0.01)			0.22	-0.02 (-0.05; 0.02)		0.32		-0.01 (-0.04; 0.02)		0.60		-0.01 (-0.04; 0.02)		0.49
**Temperament - Surgency**	0.00 (-0.04; 0.04)		0.97		0.00 (-0.03; 0.04)		0.82	0.00 (-0.03; 0.03)			0.96	0.00 (-0.03; 0.03)		0.96		0.01 (-0.03; 0.04)		0.71		0.02 (-0.02; 0.05)		0.36
**Temperament - Effortful control**	-0.02 (-0.05; 0.02)		0.36		-0.01 (-0.05; 0.03)		0.54	-0.01 (-0.04; 0.02)			0.49	-0.01 (-0.04; 0.02)		0.57		-0.01 (-0.04; 0.02)		0.73		-0.02 (-0.05; 0.01)		0.18
**Child non-verbal IQ**	-0.02 (-0.06; 0.01)		0.19		-0.01 (-0.04; 0.03)		0.60	-0.01 (-0.04; 0.02)			0.57	-0.01 (-0.04; 0.02)		0.41		0.00 (-0.03; 0.03)		0.93		-0.02 (-0.05; 0.01)		0.14
**Child self-esteem**	-0.01 (-0.04; 0.02)		0.64		0.00 (-0.03; 0.03)		0.98	0.00 (-0.03; 0.03)			0.80	0.02 (-0.01; 0.05)		0.24		0.00 (-0.02; 0.03)		0.73		0.00 (-0.03; 0.02)		0.81
Maternal sensitivity*	0.00 (-0.08; 0.08)		0.99		0.04 (-0.05; 0.13)		0.42	0.04 (-0.03; 0.12)			0.27	0.08 (0.00; 0.16)		0.04		0.07 (0.00; 0.14)		0.05		0.02 (-0.05; 0.10)		0.53
**Friendship quality**	-0.03 (-0.06; 0.01)		0.14		0.01 (-0.02; 0.05)		0.56	0.00 (-0.03; 0.04)			0.81	0.01 (-0.02; 0.04)		0.41		0.01 (-0.02; 0.04)		0.47		0.00 (-0.03; 0.03)		0.84

***MARS***																						
**Temperament**																						
Temperament – easy/difficult trait	0.06 (-0.07; 0.18)		0.35		-0.02 (-0.15; 0.12)		0.79	-0.14 (-0.25; -0.03)			0.02	-0.10 (-0.21; 0.01)		0.07		-0.05 (-0.15; 0.06)		0.37		-0.04 (-0.14; 0.05)		0.37
Temperament – self-control	0.01 (-0.10; 0.11)		0.92		0.04 (-0.07; 0.15)		0.47	-0.03 (-0.13; 0.06)			0.46	-0.03 (-0.12; 0.06)		0.49		0.00 (-0.09; 0.08)		0.92		0.01 (-0.07; 0.09)		0.84
**Child non-verbal IQ**	-0.05 (-0.16; 0.07)		0.43		0.05 (-0.07; 0.18)		0.41	0.01 (-0.09; 0.11)			0.85	-0.02 (-0.13; 0.08)		0.67		-0.03 (-0.12; 0.07)		0.56		-0.09 (-0.18; -0.01)		0.04
**Child self-esteem**	0.02 (-0.11; 0.16)		0.75		0.07 (-0.08; 0.22)		0.35	0.02 (-0.10; 0.14)			0.76	-0.06 (-0.18; 0.07)		0.37		0.09 (-0.02; 0.20)		0.11		0.01 (-0.09; 0.12)		0.78
Maternal stimulation (sensitivity)**	0.01 (-0.09; 0.11)		0.87		-0.02 (-0.12; 0.09)		0.77	-0.02 (-0.11; 0.07)			0.73	0.05 (-0.04; 0.14)		0.28		0.01 (-0.07; 0.09)		0.83		0.02 (-0.06; 0.09)		0.61

Note. Predictors included: cumulative adversity, protective factor (specific for each model), sex, total intracranial volume (only in subcortical and prefrontal regions), prenatal smoking, maternal national origin (only in Generation R), age at the MRI scan (only in Generation R), obstetric risk, and the interaction term between each protective factor and cumulative adversity. Analyses with maternal sensitivity predictors in MARS additionally adjusted for child responsiveness. Analyses with maternal sensitivity in Generation R not adjusted for maternal national origin. Negative affectivity scores in Generation R were reversed.

All brain outcomes and adversity and protective factors were standardized. Amygdala and hippocampus volumes are the mean volumes across left and right hemisphere. Abbreviations: ACC: Anterior cingulate cortex, OFC: Orbitofrontal cortex.

Generation R N = 3008. *Analyses with maternal sensitivity performed in N = 383.

MARS N = 179. **Analyses with maternal sensitivity performed in N = 173.

Adjustment for multiple testing (including all regions of interest, method: FDR): In Generation R and in MARS, no interaction survived.

At the uncorrected level, we observed three associations. First, more maternal sensitivity buffered the association of greater adversity levels with smaller right medial OFC volume in children (β = 0.08, CI 0.00 to 0.16, p_*uncorr*_ = 0.04, p_*corr*_ = 0.97) (Fig. S4). Second, easy/difficult temperament moderated the association between childhood adversity and left medial OFC volume in adults (β = − 0.14, CI − 0.25 to − 0.03, p_*uncorr*_ = 0.02, p_*corr*_ = 0.77), such that childhood adversity was associated with larger left medial OFC volumes in individuals with a more difficult temperament, and with smaller left medial OFC volumes in individuals with an easy temperament (Fig. S5). Third, a greater child IQ buffered the association between adversity and a larger right RMF in adults (β = − 0.09, CI − 0.18 to − 0.01, p_*uncorr*_ = 0.04, p_*corr*_ = 0.77) (Fig. S6).

### 3.4 Sensitivity analyses

These analyses were exploratory and thus were not adjusted for multiple testing. First, we analysed the interaction of childhood adversity and protective factors on the surface area and thickness of the cortical ROIs (Tables S6 and S7). None of these interaction effects was significant in children. In adults, maternal stimulation buffered the association between childhood adversity and smaller right medial OFC surface area (β = 0.09, CI 0.01 to 0.17, p = 0.03). This interaction was not found in children (β = 0.04, CI − 0.03 to 0.12, p = 0.26) (Fig. S7). In relation to cortical thickness, we found in adults an interaction between adversity and child IQ on the left medial OFC thickness (β = − 0.15, CI − 0.30 to − 0.01, p = 0.04), such that individuals with lower IQ had a positive association between adversity and medial OFC thickness, and individuals with higher IQ a negative association (Fig. S8). Also, there was an interaction of adversity and temperament (easy/difficult trait) on the right RMF thickness in adults (β = − 0.19, CI − 0.34 to − 0.03, p = 0.02), in which easier child temperament buffered the association of adversity with a thicker right RMF cortex (Fig. S9). Second, we examined the interaction of adversity with the protective factors separately for the left and right amygdala and hippocampus (Table S8). There was an interaction between adversity and child self-esteem on the right amygdala volume in adults (β = 0.13, CI 0.02 to 0.24, p = 0.02), such that childhood adversity was associated with smaller right amygdala in participants with low self-esteem, but with larger amygdala in participants with high self-esteem. In children, this interaction was not found (β = 0, CI − 0.04 to 0.03, p = 0.86) (Fig. S10). Third, we explored the interaction of adversity with protective factors on all main outcomes among the subsample of children with mothers of European descent in Generation R (Table S9). These analyses were performed for all protective factors, except for maternal sensitivity, as this variable was originally assessed only in mothers of Dutch national origin. Consistent with the main analyses, no interaction effects were observed in this subsample. Fourth, the principal component analyses supported a three-component solution in Generation R and in MARS (in MARS, parental death and sexual abuse were excluded because these variables had very few cases exposed), explaining 39 % of the variance of the data in Generation R and 50 % in MARS (Tables S10 and S11). The first component in both cohorts reflected social/general adverse events (mainly with loadings of early parenthood, one-parent family at child birth, unwanted pregnancy and poverty). The second component in both cohorts reflected child abuse (physical, psychological (and sexual) abuse). The third component included events that may affect the family environment, with loadings of parental psychopathology, family relationship problems, parental separation and unemployment. Analyses for the interaction of protective factors with these components revealed several interactions that were significant at an uncorrected level, and not consistent across both cohorts. Maternal sensitivity interacted with the second adversity component on the left medial OFC in children (β = 0.08, CI = 0.01–0.15). In adults, three interactions were observed: 1) for temperament (easy/difficult trait) and the first adversity component on the left medial OFC volume (β = − 0.12, CI = − 0.23 to − 0.02), 2) for temperament (self-control) and the second adversity component on the right medial OFC volume (β = − 0.13, CI = − 0.25 to − 0.01), and 3) for child IQ and the second adversity component on the hippocampal volume (β = − 0.15, CI = − 0.27 to − 0.03) (Tables S12–S14; Figs. S11–S14).

## 4 Discussion

Using two prospective birth cohorts, we investigated the moderating effects of various protective factors on the association between childhood adversity and brain morphology. Childhood adversity was associated with smaller global brain volumes in childhood, but not in adulthood. However, there was little evidence for broad brain volumetric differences associated with the interaction of adversity and the protective factors examined. Contrary to what we had expected, no interaction effect survived multiple testing correction across analyses of multiple protective factors and various ROIs. Small interaction effects observed in the childhood or adulthood outcome study only, and associations shown between the protective factors and the brain outcomes may warrant further investigation.

Childhood adversity was associated with smaller cortical grey matter, cerebral white matter and cerebellar volumes in children, but not in adults. Previous studies have described smaller cortical grey matter, white matter, and cerebellar volumes in children and adolescents exposed to early-life adversity (Bick and Nelson, 2016), as well as widespread cortical brain differences in adults (Gehred et al., 2021), although this literature is not entirely consistent and it is largely based on *severe* adverse events (Bick and Nelson, 2016). The parallel analyses of two independent cohort studies provided us with a unique opportunity to address whether childhood adversity is associated with brain structure in both late childhood and early adulthood. While the inconsistency in results could reflect initial effects of adversity on the developmental trajectories of mean brain outcomes that normalise over time, as described in some previous studies (Ellwood-Lowe et al., 2018, Rakesh et al., 2021), it is also possible that adversity effects on adult brains are more focal and not measurable with average mean brain volumes, thus requiring higher spatial precision to be detected.

In contrast to the growing research on the neurobiological outcomes of childhood adversity, the role of protective factors on this association is far less clear. While some studies have examined structural brain correlates of resilience, defining resilient individuals based on the general competence and the absence of psychopathology after being exposed to adversity (Burt et al., 2016, Eaton et al., 2022), the direct interaction between protective factors and adversity on the child brain structure has rarely been examined (Luby et al., 2019). Our results, contrary to what we hypothesised, did not provide strong evidence for any interaction effects. No interaction effects survived correction for multiple testing in the main analyses, and few uncorrected interaction effects were observed in sensitivity analyses specifically for children or adults, which warrant further investigation in larger longitudinal samples. However, these limited results are not completely unexpected. Bonanno (2021) recently described the “resilience paradox,” outlining that despite the numerous proposed protective factors, research fails to identify robust evidence for a link between protective factors and the resilience outcomes. Furthermore, this seems to hold true across distinct modelling strategies (Bonanno, 2021). One explanation for the limited evidence in our study and previous research is the lack of stability in the protective factors; that is, the dynamic nature of the protective factors may imply time-specific and context-specific effects (Bonanno, 2021). Further, the lack of strong buffering effects could suggest that protective factors do not attenuate the effect of *cumulative* adversity, but instead, may have specific buffering roles for specific adversity types (Ioannidis et al., 2020). However, similarly limited results were obtained across extensive analyses with adversity exposure modelled using principal component analyses, arguing against a strong role of the operationalization of adversity in explaining our results. Also, although useful in the search of specific effects, the analysis based on specific adverse events usually does not account for the co-occurrence of adversities and the brain morphological differences shared across exposure to different adversities (Gehred et al., 2021, Hanson et al., 2015). Additionally, although brain volumetric differences have been observed in relation to childhood adversity and, separately, to protective factors (Holz et al., 2020, McLaughlin et al., 2019), the interaction effects may be smaller, thus requiring larger neurodevelopmental samples to be detected (Maxwell and Delaney, 2004). Also, current research is largely based on standard, broad volume measures. Interaction effects may be focal (e.g., in amygdala sub-regions or on the voxel-level), and therefore not detectable with mean volumes. Furthermore, the interaction of adversity and protective factors could be more related to the degree of brain adaptability, rather than to volumetric differences per se (Shaw et al., 2006). In fact, a study demonstrated a relation between greater intellectual ability and a more plastic brain cortex (Shaw et al., 2006), and considering that resilience is often defined as the (healthy) *adjustment* to challenges (Bonanno, 2021), future studies should use repeated brain measures to determine whether resilience is reflected in the degree and characteristics of the brain *adaptability* (McEwen et al., 2015). Lastly, the small moderating effects, observed only in Generation R or MARS may also reflect a different manifestation of the interaction between adversity and protective factors across development (Eaton et al., 2022).

There was an interaction between childhood adversity and maternal sensitivity on the right medial OFC, but this result did not survive correction for multiple testing, so we recommend caution in its interpretation. Briefly, more maternal sensitivity buffered the association of greater adversity levels with smaller right medial OFC volume in children, and although not directly comparable, a similar buffering effect was observed for the right medial OFC *surface area* in adults. Although limited by a smaller sample size in Generation R (performed in a sub-cohort (N = 383)), these analyses were based on unique and robust observational sensitivity measures, prospective data collection in infants, and standardised brain morphology assessments. Previous studies in MARS have additionally suggested a protective effect of early maternal care in participants with high familial risk for psychopathology, resulting in a faster amygdala habituation, altered reward sensitivity, and fewer cases of attention deficit hyperactivity disorder (Holz et al., 2018, Holz et al., 2021). Furthermore, a potential morphometric susceptibility of the OFC to early-life adversity that may confer risk for externalising (Holz et al., 2015) and internalising psychopathology (Monninger et al., 2019) has been demonstrated. Also, neuroimaging reviews of resilience have described a link between resilience and the function and volume of the medial prefrontal cortex (Bolsinger et al., 2018, Holz et al., 2020, van der Werff et al., 2013). Since cortical neurons do not regenerate, any evidence for the buffering effect of protective factors on the association between adversity and the cortical structure likely reflects a reshaping of existing brain networks (White, 2019). Also, while some adverse events assessed in this study (e.g. physical abuse) may have been carried out by mothers, and *maternal* sensitivity may not be an actual buffer of these associations, some studies have shown that adequate maternal parenting skills could still have a positive effect on children exposed to maltreating mothers (Cicchetti et al., 2006).

Findings from additional analyses showed several associations between the protective factors examined and global brain structural differences, including a relation between child IQ and larger global brain volumes in children. There were also some regional associations for the subcortical structures, but evidence was limited for the cortical ROIs. Overall, these results could represent a promotive effect, in which the protective factors would be resources beneficial for child neuropsychological development independent of the adversity exposure (Miller-Lewis et al., 2013). Interestingly, differences in the medial OFC volume were observed in the uncorrected analyses, as well as in analyses with childhood adversity, and in interaction analyses between adversity and the protective factors. Whether these findings correspond to specific developmental trajectories and to differences in mental health outcomes needs to be elucidated by future research. Our study contributes with a preliminary view into the neuroanatomical correlates of the interplay between adversity and protective factors using mean volumes of regions-of-interest. Overall, there were no strong interaction effects despite the thorough examination of several protective factors, a rich measure of cumulative adversity, the use of data collected at multiple time points during childhood, and the reasonably large sample size. However, these findings need to be interpreted in the light of some limitations. First, our approach was based on mean volumes and regions of interest, which could limit the identification of potentially focal (e.g. voxel-wise) associations, i.e. we may have missed effects in unexamined brain regions. Second, given the early stage of the literature on protective factors, adversity and brain morphology, prior evidence was insufficient to perform a power calculation and our results should only be regarded as preliminary. However, the Generation R sample is relatively large (most previous study samples were below 150 participants). Third, despite the alignment of adversity, protective factors, and brain measures across Generation R and MARS, sample size differences and sample characteristics impeded a replication approach and limited any direct comparability of results. Fourth, information on some adversities and protective factors in Generation R were collected during the same data collection wave as the MRI, thus our results may represent cross-sectional interaction effects. Further, the data collection for adversity and protective factors was at different time points during childhood, and the effect of these time differences may have influenced some results. For instance, while some protective factors are likely to be stable across time (e.g. cognitive and temperamental characteristics (Kopala, Sibley, et al., 2018; Schneider et al., 2014)), others, such as social support, may be more variable and thus results may change depending on time of assessment. Also, short-term and long-term brain differences have been described in relation to adversities (Cortes Hidalgo et al., 2022, Gehred et al., 2021) suggesting some stability, but future research may evaluate whether buffering effects of protective factors vary across specific periods of adversity exposure. Fifth, adverse events were for the most part reported by parents (mainly mothers), which could limit the validity of our measures – for example, due to underreporting (Reuben et al., 2016). However, participants’ self-report of exposure to adversity may also be affected by limited recall of early-life events in retrospective assessments and individual characteristics (Reuben et al., 2016). Because of the limitations of both types of measurements, and the relevance of the events (e.g., sexual abuse), we included all available measures. Furthermore, we assessed mainly objective adverse events, like death or divorce, for which agreement between prospective and retrospective reports is higher compared to that of subjective events (Baldwin et al., 2019). Sixth, the analyses with the adversity components are based on a limited amount of explained variance by the adversity components, and the second component in MARS was largely based on two variables only. Finally, we acknowledge that any observed interaction effect does not imply causality. Our results may also be explained by reverse causality (i.e., brain morphology determining the protective factors and the specific role of these factors in the interaction with adversity), or by familial neurobiological features that are heritable, determine the protective factors, and are simultaneously non-randomly distributed across adversity occurrence.

Overall, our results suggest that protective factors may not modify the effect of cumulative childhood adversity, but instead, have a direct association with global and regional brain volumes. To determine the exact nature of protective effects, future neurodevelopmental studies with repeated measures of adversity, protective factors, and brain morphology should analyse the moderating effects of protective factors using brain images with higher spatial resolution, and crucially, investigate the relation to mental health outcomes.

## Data Availability

The data included in the current study are not publicly available due to legal and ethical restrictions. For access to Generation R and MARS, researchers can send their request to Vincent Jaddoe (v.jaddoe@erasmusmc.nl) and to Nathalie Holz (nathalie.holz@zi-mannheim.de), respectively.

## Funding and Acknowledgments

The authors gratefully acknowledge financial support by a short-term research grant from the 10.13039/501100001655German Academic Exchange Service (DAAD) to APCH, a ZonMw VICI grant awarded to HT (project number 016.VICI.170.200), and an award from the Netherlands Organization for Scientific Research (NWO; Spinoza prize) to MHVIJ. MJB-K was supported by the 10.13039/501100000781European Research Council (ERC AdG 669249). MHVIJ and MJB-K were also supported by the Gravitation program of the Dutch Ministry of Education, Culture, and Science and the NWO (024.001.003). SAM was supported by an Open Research Award from the Fulbright US Student Program and a Gates Cambridge Scholarship from the Gates Cambridge Trust (10.13039/100000865Bill & Melinda Gates Foundation grant OPP1144). AML acknowledges grant support by the 10.13039/501100001659German Research Foundation (DFG, Research Training Group GRK2350/1 project B02, Collaborative Research Center SFB 1158 project B09, Collaborative Research Center TRR 265 project S02, Grant ME 1591/4-1), German Federal Ministry of Education and Research (BMBF, Grants 01EF1803A, 01ZX1314G, 01GQ1003B), European Union’s Seventh Framework Programme (FP7, Grants 602450, 602805, 115300, HEALTH-F2-2010-241909), Innovative Medicines Initiative Joint Undertaking (IMI, Grant 115008) and Ministry of Science, Research and the Arts of the State of Baden-Wuerttemberg, Germany (MWK, Grant 42-04HV.MED (16)/16/1).

NEH gratefully acknowledges grants from the 10.13039/501100001659German Research Foundation (Grant nos. DFG HO 5674/2-1, GRK2350/1), the 10.13039/501100001832Radboud University (Radboud Excellence Fellowship) and the Ministry of Science, Research and the Arts of the State of Baden-Württemberg, Germany (Special support program SARS CoV-2 pandemic).

The general design of the **Generation R Study** received financial support from the Erasmus Medical Center, Rotterdam, the Erasmus University Rotterdam, ZonMw, the 10.13039/501100003246Netherlands Organisation for Scientific Research (NWO), and the Dutch Ministry of Health, Welfare and Sport. Neuroimaging and the neuroimaging infrastructure were supported by 10.13039/501100001826ZonMw TOP grant awarded to TW (Project no. 91211021). Supercomputing resources were provided by 10.13039/501100003246NWO (www.surfsara.nl, Cartesius).

The funding sources were not involved in the study design, data collection, analysis and interpretation, or in the writing of this manuscript and submission for publication.

The authors thank the contribution of children and parents, general practitioners, hospitals, midwives, and pharmacies in Rotterdam for their participation in the **Generation R Study**.

The authors thank Sibylle Heinzel, Arlette Buchmann, Dorothea Blomeyer, Erika Hohm, Katrin Zohsel, Elisabeth Reichert, Anna Becker, Angelika Bocklage, Andrea Len, Daniel Megally, and Elise Jezycki for conducting and supporting the assessments and the participants of the **MARS**.

This work was supported, in whole or in part, by the Bill & Melinda Gates Foundation [Grant OPP1144]. Under the grant conditions of the Foundation, a Creative Commons Attribution 4.0 Generic License has already been assigned to the Author Accepted Manuscript version that might arise from this submission.

## Conflicts of Interest

TB served in an advisory or consultancy role for Actelion, Hexal Pharma, Lilly, Lundbeck, Medice, Novartis and Shire. He received conference support or speaker’s fees from Lilly, Medice, Novartis and Shire. He has been involved in clinical trials conducted by Shire and Viforpharma. He received royalties from Hogrefe, Kohlhammer, CIP Medien and Oxford University Press.

AM-L has received consultant fees from the American Association for the Advancement of Science, Atheneum Partners, Blueprint Partnership, Boehringer Ingelheim, Daimler und Benz Stiftung, Elsevier, F. Hoffmann-La Roche, ICARE Schizophrenia, K. G. Jebsen Foundation, L.E.K Consulting, Lundbeck International Foundation (LINF), R. Adamczak, Roche Pharma, Science Foundation, Sumitomo Dainippon Pharma, Synapsis Foundation – Alzheimer Research Switzerland, System Analytics, and has received lectures fees including travel fees from Boehringer Ingelheim, Fama Public Relations, Institut d′investigacions Biomèdiques August Pi i Sunyer (IDIBAPS), Janssen-Cilag, Klinikum Christophsbad, Göppingen, Lilly Deutschland, Luzerner Psychiatrie, LVR Klinikum Düsseldorf, LWL Psychiatrie Verbund Westfalen-Lippe, Otsuka Pharmaceuticals, Reunions i Ciencia S. L., Spanish Society of Psychiatry, Südwestrundfunk Fernsehen, Stern TV, and Vitos Klinikum Kurhessen.

TW has received grant or research support from the Sophia Children’s Hospital Foundation, the Simons Foundation Autism Research Initiative, and the Netherlands Organisation for Health Research and Development (ZonMw). She is Editor-in-Chief for *Aperture: The Journal of the Organization for Human Brain Mapping*, and has served on the editorial board of *Neuroinformatics* and is guest editing an edition on Neuroimaging in the Global Context in *NeuroImage*.

All other authors declare no potential conflicts of interest.

## Data Availability

Data are not publicly available but requests can be made.
