# Supplementary material for: No robust evidence for an interaction between early-life adversity and protective factors on global and regional brain volumes
Source: Dev Cogn Neurosci. 2022 Oct 25;58:101166. doi: 10.1016/j.dcn.2022.101166 (PMC9636055; doi:10.1016/j.dcn.2022.101166)
Supplement: Supplementary file 2 — Supplementary material [file mmc2.docx]

**Supplementary Material for: No Robust Evidence for an Interaction Between Early-Life Adversity and Protective Factors on Global and Regional Brain Volumes**

**Methods**

**Participants**

***MARS***

From the 309 participants included in the 25-year assessment, structural brain MRI data were collected in a subsample of 201. One participant with insufficient MRI data quality and one participant with systemic lupus erythematosus were excluded from analyses. Our final study sample consisted of 179 individuals who were right-handed and who had no current psychopathology, no use of psychotropic medication, and sufficient adversity data (Figure S2).

**Measures**

***Childhood adversity***

Adversities were, for the most part, prospectively reported by parents or caregivers. A few adverse events were retrospectively reported, such as childhood physical, psychological, and sexual abuse in MARS, which were self-reported by participants at age 23 years with the brief screening version of the Childhood Trauma Questionnaire (CTQ) (Bernstein et al., 2003). Despite the retrospective nature of this questionnaire, it was included in the adversity measure given the relevance of these adverse events.

**Generation R.** Maternal marital status and whether the pregnancy was planned and/or wanted were self-reported via questionnaires during pregnancy. Maternal and paternal psychopathology were assessed at multiple time points during childhood using the depression and anxiety subscales of the Brief Symptom Inventory questionnaire (Derogatis, 1993). Poverty (yes/no) was defined based on the national low-income threshold in the Netherlands, adjusted with an equivalence factor to take into account the number of children and adults in the house and additionally adapted to the price changes over time (Centraal Bureau voor de Statistiek, 2008). Net income as well as the number of persons in the household were reported via questionnaires during pregnancy. Information on the number of children was adapted from reports collected at child age 3 and 5 years.

At age 3 years, the main caregiver reported whether the family had experienced marital problems or unemployment in the preceding two years (yes/no). Family functioning was also assessed at child age 5 and 9 years with the General Functioning subscale of the Family Assessment Device (Byles et al., 1988; Epstein et al., 1983), and the resulting sum score was dichotomized based on established cut-offs to define unhealthy family functioning (Henrichs et al., 2010). Parental separation or divorce was based on maternal reports via questionnaires at child age 3, 5, and 9 years and was classified as ever versus never occurring during childhood, as described by Xerxa et al. (2020). Data on parental death, unemployment, and physical, psychological, and sexual abuse were collected via a Life Events interview with the main caregiver when children were 9 years old. This instrument evaluates the occurrence of multiple life events during the child’s lifetime (Dunn et al., 2019), and it is based on the TRAILS study questionnaires (Amone-P'Olak et al., 2009) and the Life Events and Difficulty Schedule (Brown & Harris, 1978).

**MARS.** Information on childhood adverse events was primarily collected via three instruments. First, the Family Adversity Index is an assessment based on the enriched index defined by Rutter and Quinton (1977) (Table S1) (Holz et al., 2016). Second, information on childhood adversities was also extracted from a shortened version of the Munich Events List (MEL) (Maier-Diewald et al., 1983; Monninger et al., 2019). Third, physical, sexual, and psychological abuse were assessed with the brief screening version of the Childhood Trauma Questionnaire (CTQ) (Bernstein et al., 2003). The total score of each type of abuse was dichotomized based on previously defined cut-offs (Bevilacqua et al., 2012; Walker et al., 1999).

***Protective factors***

In MARS, child temperament was based on a parent interview and structured behavioral observations of the child at age 4.5 years (Pitzer et al., 2017). Two temperament factors were extracted: the easy-difficult trait (mainly defined by loadings of distractability/soothability, mood, approach/withdrawal, and adaptability) and self-control (based on attention/persistence, distractability/soothability, and negative loadings of activity and intensity) (Pitzer et al., 2017).

In Generation R, global self-esteem was assessed as a weighted sum score of the 18 items of the questionnaire (see also: de Lijster et al. (2019)). In MARS, child self-concept was assessed using the German version of the Perceived Competence Scales (Harter & Pike, 1984) (German version by Asendorpf and Van Aken (1993)). This measure yielded information on cognitive competencies, peer acceptance, and sports competencies (Dyer et al., 2007); the sum of the subscale scores was included in the analyses as a global measure of self-concept, referred to as self-esteem throughout the study, for ease of comparability with Generation R.

Maternal sensitivity was assessed in both cohorts by direct observation of the mothers’ behavior. In Generation R, maternal sensitivity was examined in a subsample of children of Dutch national origin (N = 383 in the pertinent analyses) during the 14-month laboratory visit. This measure was based on a stressful 8-minute psychophysiological assessment and on a 5-minute free play session, and was rated using Ainsworth’s scales (Ainsworth et al., 1974; Tharner et al., 2012). In MARS, maternal sensitivity was assessed by trained researchers that observed the interaction between the mother and the 3-month-old infant during a 10-minute semi-structured nursing and playing session. This interaction was coded using the Mannheim Rating System for Mother-Infant Interaction (Esser et al., 1989).

Friendship quality was assessed at child age 9 years in Generation R. Children rated the quality of their best friendship based on an adapted version of the Friendship Quality Questionnaire (FQQ) (Parker & Asher, 1993). The ten items (e.g., *“we tell each other secrets”*) could be rated as “*not true*,” “*somewhat true*,”, or “*very true*,” and the total score range was 10–30 (de Lijster et al., 2019).

***Brain morphology***

The reconstructed brain images in Generation R and MARS were visually inspected for quality; images with inaccuracies or artefacts were excluded from analyses (Monninger et al., 2019; Muetzel et al., 2018; Muetzel et al., 2019).

***Covariates***

**Generation R.** Information on child sex and birth weight was collected from hospital and midwife obstetric records. Prenatal smoking was self-reported by mothers during pregnancy and was categorized as “smoking during pregnancy” versus “never smoked during pregnancy.” Maternal national origin was based on the country of birth of her parents and was defined as “European descent” (including Dutch, North American, European, and Oceanian participants) versus “Other” (e.g., Surinamese, Moroccan). Low birth weight (< 2.500 g) (Rogne et al., 2017) was included as a dichotomous measure (yes/no) of obstetric risk.

**MARS.** Child sex, obstetric risk, and maternal smoking during pregnancy were assessed during a standardized interview with the parents at child age 3 months (Holz et al., 2014). Smoking was classified as “smoking during pregnancy” versus “never smoked in pregnancy.” Obstetric risk was defined as a cumulative score of the presence of nine adversities during the perinatal period, as described by Laucht et al. (2000).

**Statistical analyses**

**Sensitivity analyses.** The hemisphere-specific analyses for the amygdala and hippocampus were performed considering the functional differences between the left and right amygdala (Sergerie et al., 2008) and that the developmental trajectory of these structures has been described to differ in the left and right hemisphere (Uematsu et al., 2012).

For analyses with the adversity components in MARS, the second component was cube-root transformed because of its skewed distribution.

**Multiple testing correction.** The sensitivity analyses were not corrected for multiple testing as these were exploratory (number of tests in sensitivity analyses: analyses for the ROIs’ surface area: Generation R: 42 tests, MARS: 30 tests; analyses for the ROIs’ cortical thickness: Generation R: 42 tests, MARS: 30 tests; analyses for hemisphere-specific limbic volumes: Generation R: 28 tests, MARS: 20 tests; analyses in the European-descent subsample: Generation R: 77 tests; and analyses of interaction between protective factors and adversity components: Generation R: 231 tests, MARS: 165 tests).

**Non-response analysis**

In Generation R, we compared children in our study sample (N = 3,008) to children with adversity data but no MRI scans available (N = 2,957). We used t-tests for continuous variables and chi-squared tests for categorical ones. Children included in the analyses did not differ from those with no MRI data in maternal national origin (European descent: study sample: 66.0%, no MRI sample: 66.2%, p = 0.91) or child sex (study sample: 50.4% female, no MRI sample: 49.0% female, p = 0.29). IQ scores were higher in children in the analyses (mean (SD) = 103.0 (14.9)) compared to those with no MRI scans (mean (SD) = 100.3 (15.0), p < 0.001). The prevalence of early parenthood, psychological abuse, and physical abuse did not differ between the groups (p = 0.14, 0.80 and 0.23, respectively), whereas children without MRI scans were more likely to be exposed to poverty than those in the analyses (p = 0.003).

In MARS, we compared participants in the study sample (N = 179) with those who participated in the assessment wave in which MRI data were collected (25-year assessment) but had no MRI scans available (N = 108). We found no statistically significant difference in child sex (study sample: 58.7% female, no MRI sample: 51.9% female, p = 0.32), child IQ (mean (SD): study sample: 105.7 (11.2), no MRI sample: 104.1 (11.4), p = 0.25), prenatal maternal smoking (smoking during pregnancy: study sample: 22.3%, no MRI sample: 31.5%, p = 0.12), early parenthood (p = 0.53) and poverty (p = 0.51). Psychological abuse was more common in participants with no MRI scans available than in those included in the analyses (p = 0.01).

**Additional References**

Asendorpf, J. B., & Van Aken, M. A. (1993). Deutsche Versionen der Selbstkonzeptskalen von Harter. [German versions of Harter's self-concept scales for children.]. *Zeitschrift für Entwicklungspsychologie und Pädagogische Psychologie*, *25*(1), 64-86.

Benjamini, Y., & Hochberg, Y. (1995). Controlling the False Discovery Rate - a Practical and Powerful Approach to Multiple Testing. *Journal of the Royal Statistical Society Series B-Methodological*, *57*(1), 289-300. <Go to ISI>://WOS:A1995QE45300017

Bernstein, D. P., Stein, J. A., Newcomb, M. D., Walker, E., Pogge, D., Ahluvalia, T., Stokes, J., Handelsman, L., Medrano, M., Desmond, D., & Zule, W. (2003). Development and validation of a brief screening version of the Childhood Trauma Questionnaire. *Child abuse & neglect*, *27*(2), 169-190. <https://doi.org/https://doi.org/10.1016/S0145-2134(02)00541-0>

Bevilacqua, L., Carli, V., Sarchiapone, M., George, D. K., Goldman, D., Roy, A., & Enoch, M.-A. (2012). Interaction Between FKBP5 and Childhood Trauma and Risk of Aggressive Behavior. *Archives of General Psychiatry*, *69*(1), 62-70. <https://doi.org/10.1001/archgenpsychiatry.2011.152>

Cattell, R. B. (1960). *Culture Fair Intelligence Test, Scale 1 (Handbook)* (3rd ed.). IPAT, Champaign, IL.

Desikan, R. S., Ségonne, F., Fischl, B., Quinn, B. T., Dickerson, B. C., Blacker, D., Buckner, R. L., Dale, A. M., Maguire, R. P., Hyman, B. T., Albert, M. S., & Killiany, R. J. (2006). An automated labeling system for subdividing the human cerebral cortex on MRI scans into gyral based regions of interest. *Neuroimage*, *31*(3), 968-980. <https://doi.org/https://doi.org/10.1016/j.neuroimage.2006.01.021>

Dyer, A. S., Blomeyer, D., Laucht, M., & Schmidt, M. H. (2007). Psychische Folgen des Übergewichts im Grundschulalter. *Kindheit und Entwicklung*, *16*(3), 190-197. <https://doi.org/10.1026/0942-5403.16.3.190>

Esser, G., Scheven, A., Petrova, A., Laucht, M., & Schmidt, M. H. (1989). [The Mannheim Rating Scale for the Assessment of Mother-Child Interaction in Infancy]

Mannheimer Beurteilungsskala zur Erfassung der Mutter-Kind-Interaktion im Säuglingsalter (MBS-MKI-S). *Zeitschrift fur Kinder- und Jugendpsychiatrie*, *17*(4), 185-193.

Fischl, B. (2012). FreeSurfer. *Neuroimage*, *62*(2), 774-781. <https://doi.org/https://doi.org/10.1016/j.neuroimage.2012.01.021>

Harter, S., & Pike, R. (1984). The Pictorial Scale of Perceived Competence and Social Acceptance for Young Children. *Child Dev*, *55*(6), 1969-1982. <https://doi.org/10.2307/1129772>

Holz, N. E., Boecker-Schlier, R., Buchmann, A. F., Blomeyer, D., Jennen-Steinmetz, C., Baumeister, S., Plichta, M. M., Cattrell, A., Schumann, G., Esser, G., Schmidt, M., Buitelaar, J., Meyer-Lindenberg, A., Banaschewski, T., Brandeis, D., & Laucht, M. (2016). Ventral striatum and amygdala activity as convergence sites for early adversity and conduct disorder. *Social Cognitive and Affective Neuroscience*, *12*(2), 261-272. <https://doi.org/10.1093/scan/nsw120>

Holz, N. E., Boecker-Schlier, R., Jennen-Steinmetz, C., Hohm, E., Buchmann, A. F., Blomeyer, D., Baumeister, S., Plichta, M. M., Esser, G., Schmidt, M., Meyer-Lindenberg, A., Banaschewski, T., Brandeis, D., & Laucht, M. (2018). Early maternal care may counteract familial liability for psychopathology in the reward circuitry. *Social Cognitive and Affective Neuroscience*, *13*(11), 1191-1201. <https://doi.org/10.1093/scan/nsy087>

Holz, N. E., Boecker, R., Baumeister, S., Hohm, E., Zohsel, K., Buchmann, A. F., Blomeyer, D., Jennen-Steinmetz, C., Hohmann, S., Wolf, I., Plichta, M. M., Meyer-Lindenberg, A., Banaschewski, T., Brandeis, D., & Laucht, M. (2014). Effect of Prenatal Exposure to Tobacco Smoke on Inhibitory Control: Neuroimaging Results From a 25-Year Prospective Study. *JAMA Psychiatry*, *71*(7), 786-796. <https://doi.org/10.1001/jamapsychiatry.2014.343>

Holz, N. E., Boecker, R., Hohm, E., Zohsel, K., Buchmann, A. F., Blomeyer, D., Jennen-Steinmetz, C., Baumeister, S., Hohmann, S., Wolf, I., Plichta, M. M., Esser, G., Schmidt, M., Meyer-Lindenberg, A., Banaschewski, T., Brandeis, D., & Laucht, M. (2015). The Long-Term Impact of Early Life Poverty on Orbitofrontal Cortex Volume in Adulthood: Results from a Prospective Study Over 25 Years. *Neuropsychopharmacology*, *40*(4), 996-1004. <https://doi.org/10.1038/npp.2014.277>

Holz, N. E., Häge, A., Plichta, M. M., Boecker-Schlier, R., Jennen-Steinmetz, C., Baumeister, S., Meyer-Lindenberg, A., Laucht, M., Banaschewski, T., & Brandeis, D. (2021). Early maternal care and amygdala habituation to emotional stimuli in adulthood. *Social Cognitive and Affective Neuroscience*. <https://doi.org/10.1093/scan/nsab059>

Laucht, M., Esser, G., Baving, L., Gerhold, M., Hoesch, I., Ihle, W., Steigleider, P., Stock, B., Stoehr, R. M., Weindrich, D., & Schmidt, M. H. (2000). Behavioral Sequelae of Perinatal Insults and Early Family Adversity at 8 Years of Age. *J Am Acad Child Adolesc Psychiatry*, *39*(10), 1229-1237. <https://doi.org/https://doi.org/10.1097/00004583-200010000-00009>

Maier-Diewald, W., Wittchen, H.-U., Hecht, H., & Werner-Eilert, K. (1983). *Munich Interview for the Assessment of Life Events and Conditions - Manual*.

Monninger, M., Kraaijenvanger, E. J., Pollok, T. M., Boecker-Schlier, R., Jennen-Steinmetz, C., Baumeister, S., Esser, G., Schmidt, M., Meyer-Lindenberg, A., Laucht, M., Brandeis, D., Banaschewski, T., & Holz, N. E. (2019). The Long-Term Impact of Early Life Stress on Orbitofrontal Cortical Thickness. *Cerebral Cortex*, *30*(3), 1307-1317. <https://doi.org/10.1093/cercor/bhz167>

Pitzer, M., Esser, G., Schmidt, M. H., Hohm, E., Banaschewski, T., & Laucht, M. (2017). Child regulative temperament as a mediator of parenting in the development of depressive symptoms: a longitudinal study from early childhood to preadolescence. *Journal of Neural Transmission*, *124*(5), 631-641. <https://doi.org/10.1007/s00702-017-1682-2>

Rutter, M., & Quinton, D. (1977). Psychiatric disorder - ecological factors and concepts of causation. In M. McGurk (Ed.), *Ecological Factors in Human Development*.

Thomas, A., Chess, S., & Birch, H. G. (1968). *Temperament and behavior disorders in children*. New York U. Press.

Walker, E. A., Unutzer, J., Rutter, C., Gelfand, A., Saunders, K., VonKorff, M., Koss, M. P., & Katon, W. (1999). Costs of Health Care Use by Women HMO Members With a History of Childhood Abuse and Neglect. *Archives of General Psychiatry*, *56*(7), 609-613. <https://doi.org/10.1001/archpsyc.56.7.609>

| Table S1. Description of childhood adverse events in Generation R and MARS. | | | | |
| --- | --- | --- | --- | --- |
|  | **GENR** | | **MARS** | |
|  | **Event** | **Description of event** | **Event** | **Description of event** |
|  |  |  |  |  |
| **1** | Early parenthood | Maternal age at child birth < 21 years. Data self-reported via questionnaires during pregnancy. | Early parenthood | Family Adversity Index (FAI): Parental interview assessed in each data collection wave including 11 items, based on an "enriched" family adversity index proposed by Rutter and Quinton (1977). Item used: age of a parent <18 years at child birth or relationship between parents lasting less than 6 months at time of conception. |
| **2** | One-parent family at child birth | Pregnant mother reported having no partner. | One-parent family at child birth | FAI item: one-parent family at child birth. |
| **3** | Unwanted pregnancy | The pregnancy was unplanned and is unwanted (mixed feelings or not happy about the pregnancy). | Unwanted pregnancy | FAI item: an abortion was seriously considered. |
| **4** | Parental (maternal or paternal) psychopathology | Defined as mothers or fathers with symptoms of depression or anxiety. Assessed with the Brief Symptom Inventory (BSI) questionnaire (using the cut-off suggested by the manual). Maternal and/or paternal psychopathology were assessed at various time points during childhood (see Table 2) | Parental (maternal or paternal) psychopathology | Diagnostic interview of psychopathology in each data collection wave. Psychopathology defined as a moderate to severe disorder according to DMS III criteria. Two-stage data collection: first, a complaint inventory was used as a screening instrument. In a second stage, the Structured Clinical Interview for DSM IV was administered covering the preceding 6 months. Interviews were conducted with mothers (and in 40% of cases with fathers as well). If fathers were absent, evaluation of paternal psychopathology relied on maternal information. Assessed at multiple time points during childhood (see Table 2). |
| **5** | Poverty | Poverty during pregnancy. Poverty was defined as living below the national low-income threshold, and was based on the number of adults and children living from the same income and the monthly disposable household income (reported via questionnaire). | Poverty | Poverty at the 3-month assessment. Determined by standardized interview with the mother and defined as income level below the risk-of-poverty threshold (threshold was set to 60% of the national median equivalized disposable income adjusted for household size). |
| **6** | Death of parent | Parental death by age 9 years. Based on the Life Events Interview. Question: the child's father/mother or the other caregiver is not alive (yes/no). | Death of parent | Parental death by age 11 years. Instrument based on the Munich Events List (MEL) with data collected at multiple time points during childhood (see Table 2). Exposure to life stress was assessed in each data collection wave, with a semistructured interview to the mother or both parents until the child age of 4 years, and thereafter self-reported by parents via questionnaire. The assessment evaluates adverse life events (about all relevant areas of life stress; e.g., death of close relative, parental divorce) in the preceding 1 year. Item used: Death of a parent. |
| **7** | Family relationship problems |  | Family relationship problems | Family relationship problems by age 11 years. FAI item: low quality of partnership in two out of three areas (harmony, communication, emotional warmth). Assessed at multiple time points during childhood (see Table 2). |
|  | Problems with marriage relations | Reported at age 3 years. Question: Problems with marriage relations (yes/no) in the last 2 years. From questionnaire: Belangrijke levensgebeurtnissen [Important Life Events]. |  |  |
|  | Unhealthy family functioning | General Functioning Subscale of the Family Assessment Device. Less healthy family functioning, assessed by parental report. Reported by mothers and partners at various time points during childhood (see Table 2). |  |  |
| **8** | Parental divorce/separation | Parental divorce or separation ever occurring from child birth to age 9 years. | Parental divorce/separation | Divorce/separation by age 11 years. MEL item (e.g. assessment at 11 years): Parental separation (>3 months)/divorce. Assessed at multiple time points during childhood (see Table 2). |
| **9** | Unemployment |  | Unemployment | MEL item (e.g. assessment at 11 years): 1. Job loss, 2. Unemployment/unable to work (>3 months). Maternal and paternal unemployment assessed at multiple time points in childhood (see Table 2). |
|  | Unemployment | Reported at age 3 years. Question: Unemployment (in the family) (yes/no) in the last 2 years. From questionnaire: Belangrijke levensgebeurtnissen [Important Life Events]. |  |  |
|  | Unemployment | Reported at age 9 years. Based on the Life Events Interview. Question: Involuntary unemployment of one of the parents (yes/no) occurring from childbirth to present. |  |  |
| **10** | Physical abuse to child | Physical abuse by age 9 years. Based on the Life Events Interview. Question: Child was victim of physical violence occurring from childbirth to present. | Physical abuse to child | Assessed with the Childhood Trauma Questionnaire (CTQ). Retrospective assessment of sexual, physical, and emotional abuse, emotional and physical neglect during childhood and adolescence. The total scores for childhood sexual, physical, and emotional abuse were included in this study (see below). Cutoff points to define clinically significant abuse were based on previous studies (physical abuse = cut point of 8 or higher, sexual abuse = cut point of 8 or higher, emotional abuse = cut point of 10 or higher) (Bevilacqua et al., 2012; Walker et al., 1999). Score used: CTQ - physical abuse score. |
| **11** | Psychological abuse to child | Psychological abuse by age 9 years. Based on the Life Events Interview. Question: Someone almost used physical violence against the child and the child was scared. From childbirth to present. | Psychological abuse to child | CTQ - emotional abuse score |
| **12** | Sexual abuse | Sexual abuse by age 9 years. Based on the Life Events Interview. | Sexual abuse | CTQ - sexual abuse score |
|  | Sexual comments or movements towards the child | Question: Someone made sexual comments or movements towards the child. From childbirth to present. |  |  |
|  | Inappropriate sexual behavior | Question: Child experienced inappropriate sexual behavior. From childbirth to present. |  |  |
| All references cited here are included in the manuscript. | | | | |

| Table S2. Description of childhood protective factors in Generation R and MARS. | | | | | | |
| --- | --- | --- | --- | --- | --- | --- |
|  |  | **Generation R Study** | | **MARS** | | |
|  | **Protective factor** | **Age at assessment** | **Description** | **Protective factor** | **Age at assessment** | **Description** |
|  |  |  |  |  |  |  |
| **1** | **Temperament** |  | Assessed with the Very Short Form of the Children's Behavior Questionnaire (CBQ). Parents reported on the child reaction to events in the past 6 months. The CBQ includes three temperament dimensions: negative affectivity, surgency, and effortful control, assessed with sufficient internal consistency (Cronbach's alpha for negative affectivity: 0.75, surgency: 0.74, effortful control: 0.68; reported by Ghassabian et. al. (2014)). The three subscales were computed as the average of the respective questionnaire items in children who had less than 25% missing items per subscale. Higher scores mean greater negative affectivity, greater surgency and greater effortful control, respectively. | **Temperament** |  | Based on a parent interview and observations in two different days in both familiar and unfamiliar settings. A mean score was extracted from both interview and observations, and 9 temperament dimensions were assessed: activity, rhythmicity, approach/withdrawal, adaptability, threshold of responsiveness, intensity of reaction, the prevailing mood on a continuum between positive and negative mood, distractability/soothability, and attention span/persistence. Based on these dimensions, 2 factors were extracted: easy/difficult trait (related to mood, adaptability, etc.), and self-control (related to persistence, distractability, etc.) (for more information see: Pitzer et al., 2017). No inter-rater reliability was available due to organizational constrains, but assessments were based on highly structured protocols and all raters were carefully trained. Additionally, satisfactory inter-rater agreement was reported in a similar measure during infancy (κ = 0.82) (Pitzer et al., 2017). Higher levels in easy/difficult trait represent an easier temperament, and higher levels in self-control represent more self-control. |
|  | Temperament - Negative affectivity | 6 years | Negative affectivity subscale is defined by high positive loadings for sadness, fear, anger/frustration, and discomfort, and negative loadings from falling reactivity/soothability. This dimension is similar to neuroticism (Putnam and Rothbart et al., 2006). | Temperament - easy/difficult trait | 4.5 years |  |
|  | Temperament - Surgency | 6 years | Surgency/extraversion is characterized by impulsivity, high intensity pleasure and activity level, and negative loadings for shyness (Putnam and Rothbart et al., 2006). | Temperament - self-control |  |  |
|  | Temperament - Effortful control | 6 years | Effortful control is characterized by high inhibitory control, attentional control, low intensity pleasure, and high perceptual sensitivity. This dimension is similar to conscientiousness/constraint. |  |  |  |
| **2** | **Child non-verbal IQ** | 6 years | Assessed with two subtests of the non-verbal IQ test: SON-R 2.5-7. The reliabilities of the subtests scores used in Generation R were of 0.73 (Mosaics subtest) and 0.71 (Categories subtest) (Tellegen et. al., 1998). | **Child non-verbal IQ** | 11 years | The Culture Fair Intelligence Test-20 (CFT-20) was used to assess non-verbal cognitive abilities (non-verbal IQ test)(Cattell, 1960) |
| **3** | **Child self-esteem** | 9 years | We used an 18-item questionnaire based on the "CompetentieBelevingsSchaal voor Kinderen" (CBSK) questionnaire (Veerman et al., 1997), and the adapted question format by Wichstraum et al. (1995). The internal consistency was of ωc (categorical omega's) = 0.81 (de Lijster et. al., 2019). We computed a weighted total sum score when participants had data available for at least 13 items (allowing 28% missing values. Range of total score: 18–54). Higher scores represent greater self-esteem. | **Child self-concept** | 8 years | Assessed with the German version of the Perceived Competence Scales (Harter & Pike, 1984) (German version by Asendorpf and Van Aken (1993)). Global self-concept was defined as the sum of the subscale scores: cognitive competencies, peer acceptance, and sports competencies. The subscales' internal consistency was between α = 0.58 (sports competencies) and 0.81 (peer acceptance)(Asendorpf and Van Aken, 1993; Dyer et al., 2007). Higher scores indicated greater self-concept. |
| **4** | **Maternal sensitivity** | 14 months | Maternal sensitivity was observed in a subgroup of children of Dutch national origin (with parents and grandparents born in the Netherlands) and it was based on two observed assessments: First, mothers and children participated in an 8-min psychophysiological assessment (child's electrocardiogram measurement while watching a Teletubbies episode (BBC/Ragdoll Limited)), and then on a 5-min free play session in which the infant-mother interaction was unstructured. We used Ainsworth's rating subscales of ***cooperation*** and ***sensitivity***(Cooperation: the mother's ability to adjust her behavior in response to the infant activities, and to match cues from the infant. Sensitivity: the mother's ability to perceive and respond appropriately to the infant's signals). We calculated the maternal sensitivity score as the average of the standardized scores for both subscales in both assessments. This score was calculated for children who had at least one subscale score available, and higher scores indicated more sensitivity. The inter-coder reliability (intraclass correlation coefficient) was 0.68 (Tharner et al., 2012). | **Maternal sensitivity** | 3 months | Maternal sensitivity was assessed in a 10-minute semistructured session of nursing and playing situation (κ > 0.83) adapted from the categorical system for microanalysis of the early mother-child interaction (Jörg, et al., 1994, Holz, et al., 2018). Nine measures of mother-infant interaction behavior (e.g., vocalization, physical affect, variability) were created by coding behavior (present or absent) in 120 5s intervals using the Mannheim Rating System for Mother-Infant Interaction. **Maternal** **stimulation** (attempts to attract infant attention or establish contact (vocal, facial, or motor stimulation)) and **responsiveness** (behaviors in response to child's behaviors (vocal, facial, motor)) were coded based on the measures of mother-infant interaction. **Child** (vocal, facial, and motor) **responsiveness** was also coded. To compensate for differences in the mean between the three communication channels and to give equal weights to them, scores of vocal, facial, and motor responsiveness and stimulation were standardized and summed to create the respective **total score of maternal stimulation, responsiveness, and infant responsiveness. In these analyses, we used the measure of maternal stimulation (higher scores indicate more stimulation) to assess maternal sensitivity (based on previous evidence, e.g., Holz et al., 2018), and infant responsiveness was included as a covariate in analyses with maternal sensitivity.** |
|  |  |  |  |  |  |  |
|  |  |  |  |  |  |  |
|  | **Additional cohort-specific measures** |  |  |  |  |  |
| **5** | **Friendship Quality** | 9 years | Children's perceptions of the quality of their best friendship was assessed with a 10-item questionnaire based on the Friendship Quality Questionnaire. Items (e.g. "We give each other compliments") were rated on a 3-point Likert scale ("not true" to "very true") (Parker & Asher, 1993). The internal consistency was of ωc (categorical omega's) = 0.70 (de Lijster et. al., 2019). We computed a weighted total sum score when participants had data available for at least 8 items (allowing less than 25% missing values). Higher scores indicate a perception of greater friendship quality. | - | - | - |
|  | All references cited here are included in the manuscript. | | | | | |

| Table S3. Phi correlations between all adversity variables in Generation R. | | | | | | | | | | | | |
| --- | --- | --- | --- | --- | --- | --- | --- | --- | --- | --- | --- | --- |
|  | Early parenthood | One-parent family | Unwanted pregnancy | Parental Psychopathology | Poverty | Parental death | Family problems | Separation | Unemployment | Physical abuse | Psychological abuse | Sexual abuse |
| Early parenthood | 1 |  |  |  |  |  |  |  |  |  |  |  |
| One-parent family | 0.25 | 1 |  |  |  |  |  |  |  |  |  |  |
| Unwanted pregnancy | 0.02 | 0.15 | 1 |  |  |  |  |  |  |  |  |  |
| Parental Psychopathology | 0.13 | 0.14 | 0.05 | 1 |  |  |  |  |  |  |  |  |
| Poverty | 0.22 | 0.39 | 0.1 | 0.24 | 1 |  |  |  |  |  |  |  |
| Parental death | 0.03 | 0.02 | -0.01 | 0.02 | 0.04 | 1 |  |  |  |  |  |  |
| Family problems | 0 | 0.09 | 0.04 | 0.22 | 0.12 | 0.03 | 1 |  |  |  |  |  |
| Separation | 0.14 | 0.45 | 0.09 | 0.15 | 0.24 | 0.13 | 0.27 | 1 |  |  |  |  |
| Unemployment | 0.04 | 0.11 | 0.04 | 0.11 | 0.15 | 0 | 0.13 | 0.18 | 1 |  |  |  |
| Physical abuse | 0 | 0.05 | 0 | 0.03 | 0.04 | 0 | 0.04 | 0.04 | 0.06 | 1 |  |  |
| Psychological abuse | -0.01 | 0.03 | 0 | 0.09 | 0.02 | -0.02 | 0.08 | 0.08 | 0.11 | 0.19 | 1 |  |
| Sexual abuse | 0.01 | 0.06 | -0.01 | 0.05 | 0.04 | 0 | 0 | 0.02 | 0.07 | 0.08 | 0.11 | 1 |
| N = 3008. First imputed dataset. | |  |  |  |  |  |  |  |  |  |  |  |

| Table S4. Phi correlations between all adversity variables in MARS. | | | |  |  |  |  |  |  |  |  |  |
| --- | --- | --- | --- | --- | --- | --- | --- | --- | --- | --- | --- | --- |
|  | Early parenthood | One-parent family | Unwanted pregnancy | Parental Psychopathology | Poverty | Parental death | Family problems | Separation | Unemployment | Physical abuse | Psychological abuse | Sexual abuse |
| Early parenthood | 1 |  |  |  |  |  |  |  |  |  |  |  |
| One-parent family | 0.41 | 1 |  |  |  |  |  |  |  |  |  |  |
| Unwanted pregnancy | 0.29 | 0.38 | 1 |  |  |  |  |  |  |  |  |  |
| Parental Psychopathology | 0.23 | 0.25 | 0.20 | 1 |  |  |  |  |  |  |  |  |
| Poverty | 0.31 | 0.42 | 0.22 | 0.11 | 1 |  |  |  |  |  |  |  |
| Parental death | -0.08 | 0.11 | -0.05 | 0.13 | 0.16 | 1 |  |  |  |  |  |  |
| Family problems | 0.11 | 0.05 | 0.18 | 0.41 | 0.09 | -0.02 | 1 |  |  |  |  |  |
| Separation | 0.10 | 0.12 | 0.08 | 0.06 | 0.05 | -0.07 | 0.12 | 1 |  |  |  |  |
| Unemployment | 0.16 | 0.13 | 0.15 | 0.12 | 0.17 | -0.01 | 0.20 | -0.06 | 1 |  |  |  |
| Physical abuse | 0.13 | 0.06 | 0.13 | -0.03 | -0.08 | -0.02 | 0 | -0.02 | -0.04 | 1 |  |  |
| Psychological abuse | 0.03 | 0.06 | 0.02 | 0.03 | -0.03 | -0.04 | 0.04 | 0.04 | 0.04 | 0.34 | 1 |  |
| Sexual abuse | 0.02 | 0.11 | 0.08 | 0.05 | 0.05 | 0.32 | -0.02 | 0.13 | -0.01 | -0.02 | 0.13 | 1 |
| N = 179 |  |  |  |  |  |  |  |  |  |  |  |  |

| Table S5. Association between protective factors and brain outcomes. | | | |  |  |  |  |  |  |  |
| --- | --- | --- | --- | --- | --- | --- | --- | --- | --- | --- |
|  | *Global and regional brain outcomes* | | | | | | *Subcortical outcomes* | | | |
|  | Cortical grey matter volume | | Cerebral white matter volume | | Cerebellar volume | | Amygdala | | Hippocampus | |
|  | **β (95%CI)** | **p-value** | **β (95%CI)** | **p-value** | **β (95%CI)** | **p-value** | **β (95%CI)** | **p-value** | **β (95%CI)** | **p-value** |
| ***Generation R Study*** |  |  |  |  |  |  |  |  |  |  |
| **Temperament - Negative affectivity, reversed** | 0.05 (0.02; 0.09) | 0.003* | 0.04 (0.01; 0.08) | 0.02 | 0.01 (-0.03; 0.05) | 0.61 | -0.03 (-0.06; 0.01) | 0.10 | -0.02 (-0.06; 0.01) | 0.16 |
| **Temperament - Surgency** | -0.04 (-0.07; -0.01) | 0.02 | -0.02 (-0.05; 0.02) | 0.29 | -0.05 (-0.09; -0.02) | 0.004* | -0.06 (-0.09; -0.02) | 0.001* | -0.03 (-0.06; 0.00) | 0.07 |
| **Temperament - Effortful control** | 0.02 (-0.02; 0.05) | 0.33 | 0.02 (-0.02; 0.05) | 0.29 | 0.03 (-0.01; 0.07) | 0.09 | 0.01 (-0.02; 0.05) | 0.39 | 0.00 (-0.03; 0.03) | 0.91 |
| **Child non-verbal IQ** | 0.13 (0.10; 0.17) | <0.001* | 0.12 (0.08; 0.15) | <0.001* | 0.10 (0.06; 0.13) | <0.001* | 0.02 (-0.01; 0.05) | 0.25 | 0.02 (-0.01; 0.05) | 0.16 |
| **Child self-esteem** | 0.05 (0.01; 0.08) | 0.01 | 0.05 (0.02; 0.09) | 0.003* | 0.04 (0.00; 0.08) | 0.03 | -0.04 (-0.07; -0.01) | 0.004* | -0.02 (-0.05; 0.01) | 0.15 |
| **Maternal sensitivity*** | 0.00 (-0.09; 0.09) | 0.98 | 0.00 (-0.09; 0.09) | 0.98 | -0.04 (-0.13; 0.05) | 0.37 | 0.02 (-0.06; 0.09) | 0.66 | 0.02 (-0.06; 0.10) | 0.63 |
| **Friendship quality** | 0.01 (-0.03; 0.05) | 0.54 | 0.02 (-0.02; 0.06) | 0.26 | 0.04 (0.00; 0.07) | 0.06 | -0.02 (-0.06; 0.01) | 0.14 | -0.03 (-0.07; 0.00) | 0.04 |
| ***MARS*** |  |  |  |  |  |  |  |  |  |  |
| **Temperament** |  |  |  |  |  |  |  |  |  |  |
| Temperament - easy/difficult trait | -0.05 (-0.17; 0.06) | 0.36 | -0.07 (-0.19; 0.05) | 0.25 | -0.03 (-0.15; 0.09) | 0.61 | -0.04 (-0.14; 0.05) | 0.36 | -0.02 (-0.14; 0.09) | 0.70 |
| Temperament - self-control | 0.03 (-0.09; 0.14) | 0.66 | -0.01 (-0.13; 0.12) | 0.91 | 0.05 (-0.07; 0.17) | 0.41 | 0.03 (-0.06; 0.13) | 0.51 | -0.01 (-0.13; 0.10) | 0.84 |
| **Child non-verbal IQ** | 0.12 (0.01; 0.23) | 0.03 | 0.18 (0.06; 0.30) | 0.003 | 0.13 (0.01; 0.25) | 0.03 | 0.06 (-0.03; 0.16) | 0.19 | 0.12 (0.01; 0.24) | 0.03 |
| **Child self-esteem** | 0.05 (-0.06; 0.17) | 0.35 | 0.03 (-0.09; 0.15) | 0.64 | 0.08 (-0.04; 0.20) | 0.20 | -0.07 (-0.17; 0.02) | 0.14 | -0.12 (-0.23; -0.01) | 0.03 |
| **Maternal stimulation (sensitivity)**** | -0.05 (-0.17; 0.06) | 0.37 | -0.12 (-0.24; 0.00) | 0.06 | -0.08 (-0.20; 0.05) | 0.22 | -0.05 (-0.15; 0.05) | 0.32 | -0.03 (-0.14; 0.09) | 0.66 |
| Note. Predictors included: the protective factor (specific for each model), sex, total intracranial volume (only in subcortical and prefrontal regions), maternal national origin (only in Generation R), age at the MRI scan (only in Generation R). Analyses with maternal sensitivity predictors in MARS additionally adjusted for child responsiveness. Analyses with maternal sensitivity in Generation R not adjusted for maternal national origin. Negative affectivity scores in Generation R were reversed. | | | | | | | | | | |
| All brain outcomes and protective factors were standardized. Amygdala and hippocampus volumes are the mean volumes across left and right hemisphere. Abbreviations: ACC: Anterior cingulate cortex, OFC: Orbitofrontal cortex. | | | | | | | | | | |
| Generation R N = 3,008. *Analyses with maternal sensitivity performed in N = 383. | | | | | | | | | | |
| MARS N = 179. **Analyses with maternal sensitivity performed in N = 173. | | | | | | | | | | |
| * p-values that survived adjustment for multiple testing (including all regions of interest, method: FDR). No p-value survived correction in MARS. | | | | | | | | | | |

| Table S5. Association between protective factors and brain outcomes *(cont.)* | | | | | | | | |  |  |  |  |
| --- | --- | --- | --- | --- | --- | --- | --- | --- | --- | --- | --- | --- |
|  | Left ACC | | Right ACC | | Left medial OFC | | Right medial OFC | | Left rostral middle frontal volume | | Right rostral middle frontal volume | |
|  | **β (95%CI)** | **p-value** | **β (95%CI)** | **p-value** | **β (95%CI)** | **p-value** | **β (95%CI)** | **p-value** | **β (95%CI)** | **p-value** | **β (95%CI)** | **p-value** |
| ***Generation R Study*** |  |  |  |  |  |  |  |  |  |  |  |  |
| **Temperament - Negative affectivity, reversed** | 0.01 (-0.02; 0.04) | 0.52 | 0.01 (-0.03; 0.05) | 0.60 | 0.01 (-0.03; 0.04) | 0.62 | -0.02 (-0.05; 0.01) | 0.27 | 0.00 (-0.03; 0.03) | 0.93 | -0.01 (-0.04; 0.02) | 0.71 |
| **Temperament - Surgency** | 0.00 (-0.03; 0.04) | 0.90 | -0.01 (-0.05; 0.03) | 0.58 | -0.01 (-0.04; 0.02) | 0.60 | -0.01 (-0.04; 0.02) | 0.61 | -0.03 (-0.06; 0.00) | 0.054 | -0.02 (-0.05; 0.01) | 0.29 |
| **Temperament - Effortful control** | -0.01 (-0.05; 0.02) | 0.39 | -0.02 (-0.06; 0.02) | 0.28 | -0.01 (-0.05; 0.02) | 0.49 | 0.03 (-0.01; 0.06) | 0.13 | -0.01 (-0.04; 0.02) | 0.42 | -0.01 (-0.04; 0.02) | 0.51 |
| **Child non-verbal IQ** | 0.00 (-0.03; 0.03) | 0.97 | 0.01 (-0.02; 0.05) | 0.45 | -0.01 (-0.05; 0.02) | 0.42 | 0.02 (-0.02; 0.05) | 0.34 | 0.00 (-0.03; 0.03) | 0.82 | 0.01 (-0.02; 0.04) | 0.40 |
| **Child self-esteem** | -0.01 (-0.04; 0.03) | 0.69 | -0.01 (-0.04; 0.03) | 0.60 | 0.00 (-0.04; 0.03) | 0.85 | 0.01 (-0.03; 0.04) | 0.72 | 0.01 (-0.02; 0.04) | 0.55 | 0.02 (-0.01; 0.05) | 0.31 |
| **Maternal sensitivity*** | 0.03 (-0.05; 0.12) | 0.48 | 0.05 (-0.04; 0.15) | 0.25 | 0.09 (0.01; 0.17) | 0.02 | 0.07 (-0.02; 0.15) | 0.11 | 0.01 (-0.06; 0.08) | 0.75 | 0.05 (-0.03; 0.12) | 0.23 |
| **Friendship quality** | 0.03 (-0.01; 0.06) | 0.16 | 0.03 (-0.01; 0.07) | 0.11 | -0.02 (-0.05; 0.02) | 0.27 | 0.00 (-0.03; 0.03) | 0.98 | 0.01 (-0.02; 0.04) | 0.38 | 0.02 (-0.01; 0.06) | 0.12 |
| ***MARS*** |  |  |  |  |  |  |  |  |  |  |  |  |
| **Temperament** |  |  |  |  |  |  |  |  |  |  |  |  |
| Temperament - easy/difficult trait | -0.1 (-0.22; 0.02) | 0.09 | 0.05 (-0.08; 0.17) | 0.47 | 0.01 (-0.10; 0.12) | 0.84 | -0.04 (-0.14; 0.07) | 0.51 | -0.08 (-0.18; 0.02) | 0.13 | -0.05 (-0.14; 0.04) | 0.30 |
| Temperament - self-control | 0.05 (-0.07; 0.17) | 0.43 | -0.07 (-0.20; 0.06) | 0.29 | -0.08 (-0.18; 0.03) | 0.17 | 0.04 (-0.06; 0.15) | 0.42 | -0.02 (-0.12; 0.08) | 0.65 | 0.00 (-0.09; 0.09) | 0.99 |
| **Child non-verbal IQ** | 0.00 (-0.12; 0.12) | 0.98 | -0.01 (-0.13; 0.12) | 0.93 | 0.07 (-0.04; 0.18) | 0.22 | 0.00 (-0.10; 0.11) | 0.94 | -0.05 (-0.15; 0.05) | 0.36 | -0.05 (-0.14; 0.05) | 0.32 |
| **Child self-esteem** | -0.04 (-0.16; 0.08) | 0.52 | 0.02 (-0.11; 0.14) | 0.80 | 0.04 (-0.07; 0.15) | 0.46 | -0.02 (-0.12; 0.09) | 0.74 | 0.04 (-0.06; 0.14) | 0.46 | 0.08 (-0.01; 0.17) | 0.09 |
| **Maternal stimulation (sensitivity)**** | 0.04 (-0.08; 0.16) | 0.55 | -0.06 (-0.18; 0.07) | 0.39 | -0.02 (-0.13; 0.09) | 0.76 | 0.04 (-0.07; 0.15) | 0.50 | -0.06 (-0.16; 0.04) | 0.24 | 0.00 (-0.10; 0.09) | 0.95 |
| Note. Predictors included: the protective factor (specific for each model), sex, total intracranial volume (only in subcortical and prefrontal regions), maternal national origin (only in Generation R), age at the MRI scan (only in Generation R). Analyses with maternal sensitivity predictors in MARS additionally adjusted for child responsiveness. Analyses with maternal sensitivity in Generation R not adjusted for maternal national origin. Negative affectivity scores in Generation R were reversed. | | | | | | | | | | | | |
| All brain outcomes and protective factors were standardized. Amygdala and hippocampus volumes are the mean volumes across left and right hemisphere. Abbreviations: ACC: Anterior cingulate cortex, OFC: Orbitofrontal cortex. | | | | | | | | | | | | |
| Generation R N = 3,008. *Analyses with maternal sensitivity performed in N = 383. | | | | | | | | | | | | |
| MARS N = 179. **Analyses with maternal sensitivity performed in N = 173. | | | | | | | | | | | | |
| * p-values that survived adjustment for multiple testing (including all regions of interest, method: FDR). No p-value survived correction in MARS. | | | | | | | | | | | | |

| Table S6. Interaction between protective factors and childhood adversity on the **surface area** of the cortical regions of interest. | | | | | | | | | | | | |
| --- | --- | --- | --- | --- | --- | --- | --- | --- | --- | --- | --- | --- |
|  | Left ACC | | Right ACC | | Left medial OFC | | Right medial OFC | | Left rostral middle frontal cortex | | Right rostral middle frontal cortex | |
|  | **β (95%CI)** | **p-value** | **β (95%CI)** | **p-value** | **β (95%CI)** | **p-value** | **β (95%CI)** | **p-value** | **β (95%CI)** | **p-value** | **β (95%CI)** | **p-value** |
| ***Generation R Study*** |  |  |  |  |  |  |  |  |  |  |  |  |
|  |  |  |  |  |  |  |  |  |  |  |  |  |
| **Temperament - Negative affectivity, reversed** | 0.00 (-0.03; 0.03) | 0.98 | -0.01 (-0.05; 0.02) | 0.44 | -0.01 (-0.04; 0.03) | 0.75 | 0.00 (-0.03; 0.03) | 0.88 | 0.00 (-0.03; 0.03) | 0.77 | -0.01 (-0.04; 0.02) | 0.62 |
| **Temperament - Surgency** | 0.01 (-0.03; 0.04) | 0.75 | 0.01 (-0.03; 0.04) | 0.74 | 0.00 (-0.03; 0.03) | 0.94 | -0.01 (-0.04; 0.02) | 0.65 | 0.00 (-0.03; 0.03) | 0.98 | 0.01 (-0.03; 0.04) | 0.71 |
| **Temperament - Effortful control** | -0.01 (-0.05; 0.02) | 0.39 | 0.00 (-0.04; 0.03) | 0.90 | -0.02 (-0.05; 0.01) | 0.12 | -0.02 (-0.04; 0.01) | 0.28 | -0.01 (-0.04; 0.02) | 0.59 | -0.02 (-0.04; 0.01) | 0.23 |
| **Child non-verbal IQ** | -0.03 (-0.06; 0.01) | 0.11 | -0.01 (-0.04; 0.02) | 0.51 | -0.01 (-0.04; 0.02) | 0.52 | -0.01 (-0.04; 0.02) | 0.48 | 0.00 (-0.02; 0.03) | 0.74 | -0.02 (-0.04; 0.01) | 0.27 |
| **Child self-esteem** | -0.01 (-0.04; 0.02) | 0.44 | 0.00 (-0.03; 0.04) | 0.77 | 0.01 (-0.02; 0.04) | 0.36 | 0.01 (-0.02; 0.04) | 0.38 | 0.01 (-0.02; 0.03) | 0.58 | 0.00 (-0.03; 0.03) | 0.92 |
| **Maternal sensitivity*** | 0.01 (-0.06; 0.09) | 0.71 | 0.03 (-0.06; 0.11) | 0.54 | 0.01 (-0.06; 0.09) | 0.73 | 0.04 (-0.03; 0.12) | 0.26 | 0.06 (-0.01; 0.13) | 0.08 | 0.01 (-0.06; 0.08) | 0.72 |
| **Friendship quality** | -0.02 (-0.05; 0.01) | 0.23 | 0.01 (-0.03; 0.04) | 0.70 | 0.01 (-0.03; 0.04) | 0.71 | 0.00 (-0.03; 0.02) | 0.76 | 0.01 (-0.02; 0.04) | 0.63 | 0.00 (-0.03; 0.03) | 0.85 |
| ***MARS*** |  |  |  |  |  |  |  |  |  |  |  |  |
| **Temperament** |  |  |  |  |  |  |  |  |  |  |  |  |
| Temperament - easy/difficult trait | 0.08 (-0.03; 0.20) | 0.16 | 0.00 (-0.13; 0.12) | 0.98 | -0.07 (-0.18; 0.04) | 0.24 | -0.03 (-0.14; 0.07) | 0.53 | 0.00 (-0.10; 0.10) | 0.93 | 0.03 (-0.06; 0.12) | 0.46 |
| Temperament - self-control | 0.01 (-0.08; 0.11) | 0.77 | 0.03 (-0.08; 0.13) | 0.58 | 0.01 (-0.08; 0.10) | 0.81 | 0.03 (-0.06; 0.11) | 0.52 | 0.03 (-0.06; 0.11) | 0.51 | 0.02 (-0.05; 0.10) | 0.52 |
| **Child non-verbal IQ** | -0.01 (-0.12; 0.10) | 0.83 | 0.05 (-0.06; 0.17) | 0.36 | 0.08 (-0.02; 0.18) | 0.13 | 0.04 (-0.06; 0.14) | 0.41 | -0.01 (-0.10; 0.08) | 0.82 | -0.04 (-0.13; 0.04) | 0.30 |
| **Child self-esteem** | 0.04 (-0.09; 0.17) | 0.54 | 0.08 (-0.05; 0.22) | 0.23 | 0.04 (-0.08; 0.17) | 0.49 | -0.02 (-0.13; 0.10) | 0.78 | 0.09 (-0.02; 0.20) | 0.11 | 0.04 (-0.06; 0.14) | 0.42 |
| **Maternal stimulation (sensitivity)**** | 0.05 (-0.04; 0.14) | 0.31 | 0.00 (-0.10; 0.10) | 0.94 | 0.02 (-0.06; 0.11) | 0.59 | 0.09 (0.01; 0.17) | 0.03 | 0.05 (-0.03; 0.13) | 0.22 | 0.05 (-0.02; 0.12) | 0.14 |
| Predictors included: cumulative adversity, protective factor (specific for each model), (child) sex, total intracranial volume, prenatal smoking, maternal national origin (only in Generation R), (child) age at the MRI scan (only in Generation R), obstetric risk, and the interaction term between each protective factor and cumulative adversity. Analyses with maternal sensitivity in MARS additionally adjusted for child responsiveness. Analyses with maternal sensitivity in Generation R not adjusted for national origin. Negative affectivity scores in Generation R were reversed. | | | | | | | | | | | | |
| All brain outcomes and adversity and protective factors were standardized. Abbreviations: ACC: Anterior cingulate cortex, OFC: Orbitofrontal cortex | | | | | | | | | | | | |
| Generation R N = 3,008. *Analyses with maternal sensitivity in N = 383. | | | | | | | | | | | | |
| MARS N = 179. **Analyses with maternal sensitivity in N = 173. | | | | | | | | | | | | |

| Table S7. Interaction between protective factors and childhood adversity on the **thickness** of the cortical regions of interest. | | | | | | | | | | |  |  |
| --- | --- | --- | --- | --- | --- | --- | --- | --- | --- | --- | --- | --- |
|  | Left ACC | | Right ACC | | Left medial OFC | | Right medial OFC | | Left rostral middle frontal cortex | | Right rostral middle frontal cortex | |
|  | **β (95%CI)** | **p-value** | **β (95%CI)** | **p-value** | **β (95%CI)** | **p-value** | **β (95%CI)** | **p-value** | **β (95%CI)** | **p-value** | **β (95%CI)** | **p-value** |
| ***Generation R Study*** |  |  |  |  |  |  |  |  |  |  |  |  |
|  |  |  |  |  |  |  |  |  |  |  |  |  |
| **Temperament - Negative affectivity, reversed** | 0.01 (-0.03; 0.05) | 0.56 | -0.04 (-0.08; 0.01) | 0.09 | -0.03 (-0.07; 0.01) | 0.17 | -0.03 (-0.07; 0.01) | 0.15 | -0.02 (-0.06; 0.02) | 0.31 | -0.01 (-0.05; 0.03) | 0.67 |
| **Temperament - Surgency** | 0 (-0.05; 0.04) | 0.85 | 0.01 (-0.04; 0.05) | 0.78 | 0 (-0.04; 0.05) | 0.82 | 0.02 (-0.02; 0.07) | 0.28 | 0.02 (-0.02; 0.07) | 0.36 | 0.03 (-0.01; 0.07) | 0.19 |
| **Temperament - Effortful control** | -0.01 (-0.05; 0.03) | 0.58 | -0.01 (-0.05; 0.03) | 0.57 | 0.03 (-0.01; 0.07) | 0.18 | 0.02 (-0.02; 0.06) | 0.35 | 0 (-0.03; 0.04) | 0.81 | 0 (-0.04; 0.04) | 0.95 |
| **Child non-verbal IQ** | 0.01 (-0.03; 0.04) | 0.79 | 0.01 (-0.03; 0.04) | 0.79 | -0.02 (-0.06; 0.02) | 0.41 | 0 (-0.04; 0.04) | 0.84 | -0.01 (-0.06; 0.03) | 0.48 | -0.01 (-0.05; 0.03) | 0.52 |
| **Child self-esteem** | 0.01 (-0.02; 0.05) | 0.47 | -0.02 (-0.06; 0.02) | 0.39 | -0.02 (-0.06; 0.01) | 0.22 | 0.01 (-0.03; 0.05) | 0.58 | 0.01 (-0.03; 0.05) | 0.64 | 0 (-0.03; 0.04) | 0.81 |
| **Maternal sensitivity*** | 0 (-0.1; 0.1) | 0.98 | 0.03 (-0.07; 0.13) | 0.56 | 0.03 (-0.06; 0.13) | 0.49 | 0.06 (-0.03; 0.16) | 0.19 | 0.02 (-0.08; 0.11) | 0.72 | 0.04 (-0.06; 0.13) | 0.46 |
| **Friendship quality** | 0 (-0.04; 0.04) | 0.95 | 0 (-0.04; 0.04) | 0.87 | 0 (-0.05; 0.04) | 0.82 | 0.04 (0; 0.08) | 0.05 | 0.01 (-0.03; 0.05) | 0.57 | 0.01 (-0.03; 0.05) | 0.60 |
| ***MARS*** |  |  |  |  |  |  |  |  |  |  |  |  |
| **Temperament** |  |  |  |  |  |  |  |  |  |  |  |  |
| Temperament - easy/difficult trait | -0.08 (-0.24; 0.07) | 0.30 | -0.05 (-0.21; 0.11) | 0.55 | -0.13 (-0.29; 0.02) | 0.09 | -0.12 (-0.28; 0.03) | 0.12 | -0.14 (-0.29; 0.02) | 0.09 | -0.19 (-0.34; -0.03) | 0.02 |
| Temperament - self-control | -0.04 (-0.17; 0.09) | 0.58 | 0.04 (-0.09; 0.17) | 0.56 | -0.13 (-0.26; 0.00) | 0.05 | -0.05 (-0.18; 0.08) | 0.45 | -0.05 (-0.18; 0.08) | 0.44 | -0.04 (-0.17; 0.09) | 0.58 |
| **Child non-verbal IQ** | -0.06 (-0.21; 0.09) | 0.42 | -0.05 (-0.20; 0.09) | 0.49 | -0.15 (-0.30; -0.01) | 0.04 | -0.04 (-0.19; 0.10) | 0.58 | 0.01 (-0.14; 0.16) | 0.90 | -0.11 (-0.26; 0.03) | 0.12 |
| **Child self-esteem** | 0.00 (-0.17; 0.17) | 1.00 | 0.00 (-0.18; 0.17) | 0.96 | 0.00 (-0.17; 0.17) | 0.96 | -0.07 (-0.24; 0.10) | 0.41 | 0.03 (-0.14; 0.20) | 0.70 | -0.09 (-0.26; 0.08) | 0.29 |
| **Maternal stimulation (sensitivity)**** | -0.10 (-0.23; 0.02) | 0.10 | 0.02 (-0.10; 0.15) | 0.72 | -0.06 (-0.19; 0.06) | 0.31 | -0.07 (-0.20; 0.05) | 0.24 | -0.06 (-0.19; 0.06) | 0.31 | -0.06 (-0.18; 0.07) | 0.38 |
| Predictors included: cumulative adversity, protective factor (specific for each model), (child) sex, prenatal smoking, maternal national origin (only in Generation R), (child) age at the MRI scan (only in Generation R), obstetric risk, and the interaction term between each protective factor and cumulative adversity. Analyses with maternal sensitivity in MARS additionally adjusted for child responsiveness. Analyses with maternal sensitivity in Generation R not adjusted for national origin. Negative affectivity scores in Generation R were reversed. | | | | | | | | | | | | |
| All brain outcomes and adversity and protective factors were standardized. Abbreviations: ACC: Anterior cingulate cortex, OFC: Orbitofrontal cortex | | | | | | | | | | |  |  |
| Generation R N = 3,008. *Analyses with maternal sensitivity in N = 383. | | | |  |  |  |  |  |  |  |  |  |
| MARS N = 179. **Analyses with maternal sensitivity in N = 173. | | |  |  |  |  |  |  |  |  |  |  |

| Table S8. Interaction between protective factors and childhood adversity on the **left and right** amygdala and hippocampus. | | | | | | | | |
| --- | --- | --- | --- | --- | --- | --- | --- | --- |
|  | *Amygdala* | | | | *Hippocampus* | | | |
|  | Left | | Right | | Left | | Right | |
|  | **β (95%CI)** | **p-value** | **β (95%CI)** | **p-value** | **β (95%CI)** | **p-value** | **β (95%CI)** | **p-value** |
| ***Generation R Study*** |  |  |  |  |  |  |  |  |
| **Temperament - Negative affectivity, reversed** | -0.01 (-0.04; 0.03) | 0.75 | 0.00 (-0.03; 0.03) | 0.90 | -0.01 (-0.05; 0.02) | 0.41 | -0.01 (-0.04; 0.02) | 0.52 |
| **Temperament - Surgency** | 0.02 (-0.02; 0.05) | 0.30 | 0.01 (-0.03; 0.04) | 0.72 | -0.01 (-0.04; 0.03) | 0.65 | -0.01 (-0.04; 0.02) | 0.57 |
| **Temperament - Effortful control** | 0.01 (-0.03; 0.05) | 0.59 | 0.00 (-0.04; 0.03) | 0.95 | 0.02 (-0.01; 0.05) | 0.21 | 0.02 (-0.01; 0.05) | 0.27 |
| **Child non-verbal IQ** | -0.01 (-0.04; 0.02) | 0.54 | -0.02 (-0.05; 0.02) | 0.34 | -0.01 (-0.05; 0.02) | 0.41 | -0.02 (-0.05; 0.02) | 0.30 |
| **Child self-esteem** | 0.00 (-0.03; 0.04) | 0.77 | 0.00 (-0.04; 0.03) | 0.86 | 0.01 (-0.02; 0.04) | 0.59 | 0.00 (-0.03; 0.04) | 0.76 |
| **Maternal sensitivity*** | 0.03 (-0.04; 0.11) | 0.40 | 0.06 (-0.02; 0.14) | 0.12 | 0.00 (-0.08; 0.07) | 0.97 | 0.01 (-0.07; 0.09) | 0.80 |
| **Friendship quality** | -0.02 (-0.06; 0.01) | 0.15 | -0.02 (-0.05; 0.01) | 0.27 | -0.01 (-0.05; 0.02) | 0.38 | -0.01 (-0.05; 0.02) | 0.42 |
| ***MARS*** |  |  |  |  |  |  |  |  |
| **Temperament** |  |  |  |  |  |  |  |  |
| Temperament - easy/difficult trait | -0.04 (-0.15; 0.07) | 0.49 | -0.04 (-0.14; 0.07) | 0.48 | -0.04 (-0.16; 0.07) | 0.48 | -0.04 (-0.15; 0.08) | 0.55 |
| Temperament - self-control | 0.02 (-0.07; 0.11) | 0.64 | -0.02 (-0.10; 0.07) | 0.70 | -0.01 (-0.11; 0.08) | 0.79 | -0.01 (-0.11; 0.09) | 0.90 |
| **Child non-verbal IQ** | -0.07 (-0.17; 0.03) | 0.18 | -0.09 (-0.18; 0) | 0.06 | -0.02 (-0.13; 0.08) | 0.66 | 0.01 (-0.10; 0.12) | 0.83 |
| **Child self-esteem** | 0.06 (-0.06; 0.18) | 0.30 | 0.13 (0.02; 0.24) | 0.02 | 0.08 (-0.04; 0.21) | 0.18 | 0.08 (-0.04; 0.21) | 0.19 |
| **Maternal stimulation (sensitivity)**** | 0.04 (-0.05; 0.13) | 0.42 | -0.01 (-0.09; 0.07) | 0.79 | 0.03 (-0.06; 0.12) | 0.53 | 0.03 (-0.06; 0.13) | 0.48 |
| Predictors included: cumulative adversity, protective factor (specific for each model), (child) sex, total intracranial volume, prenatal smoking, maternal national origin (only in Generation R), (child) age at the MRI scan (only in Generation R), obstetric risk, and the interaction term between each protective factor and cumulative adversity. Analyses with maternal sensitivity in MARS additionally adjusted for child responsiveness. Analyses with maternal sensitivity in Generation R not adjusted for national origin. Negative affectivity scores in Generation R were reversed. | | | | | | | | |
| All brain outcomes and adversity and protective factors were standardized. | | | | | | | | |
| Generation R N = 3,008. *Analyses with maternal sensitivity in N=383. | | | | | | | | |
| MARS N = 179. **Analyses with maternal sensitivity in N = 173. | |  |  |  |  |  |  |  |

| Table S9. Interaction between protective factors and childhood adversity in relation to brain outcomes in children **with mothers of European descent.** | | | | | | | | | |  |
| --- | --- | --- | --- | --- | --- | --- | --- | --- | --- | --- |
|  | *Global and regional brain outcomes* | | | | | | *Subcortical outcomes* | | | |
|  | Cortical grey matter volume | | Cerebral white matter volume | | Cerebellar volume | | Amygdala | | Hippocampus | |
|  | **β (95%CI)** | **p-value** | **β (95%CI)** | **p-value** | **β (95%CI)** | **p-value** | **β (95%CI)** | **p-value** | **β (95%CI)** | **p-value** |
| ***Generation R Study*** |  |  |  |  |  |  |  |  |  |  |
| **Temperament - Negative affectivity, reversed** | 0.00 (-0.04; 0.05) | 0.91 | 0.01 (-0.03; 0.05) | 0.70 | 0.04 (-0.01; 0.08) | 0.09 | 0.00 (-0.03; 0.04) | 0.81 | -0.01 (-0.05; 0.03) | 0.63 |
| **Temperament - Surgency** | 0.02 (-0.03; 0.06) | 0.44 | 0.00 (-0.05; 0.04) | 0.92 | 0.03 (-0.01; 0.08) | 0.17 | 0.02 (-0.02; 0.06) | 0.31 | 0.00 (-0.04; 0.04) | 0.95 |
| **Temperament - Effortful control** | -0.02 (-0.06; 0.02) | 0.36 | 0.00 (-0.04; 0.04) | 0.94 | 0.00 (-0.04; 0.04) | 0.91 | 0.02 (-0.02; 0.05) | 0.42 | 0.02 (-0.02; 0.05) | 0.35 |
| **Child non-verbal IQ** | 0.02 (-0.02; 0.06) | 0.35 | 0.00 (-0.04; 0.04) | 0.97 | 0.01 (-0.04; 0.05) | 0.80 | 0.00 (-0.04; 0.04) | 0.99 | -0.02 (-0.06; 0.01) | 0.19 |
| **Child self-esteem** | 0.00 (-0.04; 0.04) | 0.98 | 0.01 (-0.03; 0.05) | 0.61 | -0.01 (-0.05; 0.03) | 0.78 | 0.00 (-0.04; 0.03) | 0.88 | 0.00 (-0.04; 0.03) | 0.87 |
| **Maternal sensitivity** | - | - | - | - | - | - | - | - | - | - |
| **Friendship quality** | 0.00 (-0.04; 0.04) | 0.94 | 0.02 (-0.02; 0.06) | 0.31 | 0.04 (0.00; 0.08) | 0.08 | -0.03 (-0.06; 0.01) | 0.21 | -0.03 (-0.06; 0.01) | 0.21 |
| Note. Predictors included: cumulative adversity, protective factor (specific for each model), (child) sex, total intracranial volume (only in subcortical outcomes), prenatal smoking, (child) age at the MRI scan, obstetric risk, and the interaction term between each protective factor and cumulative adversity. For the results of maternal sensitivity in Generation R see the main analyses. Negative affectivity scores in Generation R were reversed. | | | | | | | | | | |
| All brain outcomes and adversity and protective factors were standardized. Amygdala and hippocampus volumes are the mean volumes across left and right hemisphere. | | | | | | | | | | |
| Generation R N=1,947 | | | | | | | | | | |

| Table S9. Interaction between protective factors and childhood adversity in relation to brain outcomes in children with **mothers of European descent.** *(cont.)* | | | | | | | | | | | | | |
| --- | --- | --- | --- | --- | --- | --- | --- | --- | --- | --- | --- | --- | --- |
|  | Left ACC | | Right ACC | | Left medial OFC | | Right medial OFC | | Left rostral middle frontal cortex | | Right rostral middle frontal cortex | |  |
|  | **β (95%CI)** | **p-value** | **β (95%CI)** | **p-value** | **β (95%CI)** | **p-value** | **β (95%CI)** | **p-value** | **β (95%CI)** | **p-value** | **β (95%CI)** | **p-value** |  |
| ***Generation R Study*** |  |  |  |  |  |  |  |  |  |  |  |  |  |
| **Temperament - Negative affectivity, reversed** | 0.01 (-0.03; 0.04) | 0.77 | -0.03 (-0.07; 0.01) | 0.14 | -0.02 (-0.07; 0.02) | 0.23 | -0.02 (-0.05; 0.02) | 0.42 | -0.01 (-0.04; 0.03) | 0.69 | -0.01 (-0.04; 0.03) | 0.70 |  |
| **Temperament - Surgency** | 0.00 (-0.04; 0.04) | 0.97 | 0.01 (-0.04; 0.05) | 0.75 | 0.01 (-0.03; 0.05) | 0.66 | 0.00 (-0.04; 0.04) | 0.95 | 0.00 (-0.03; 0.04) | 0.88 | 0.00 (-0.04; 0.04) | 0.85 |  |
| **Temperament - Effortful control** | -0.01 (-0.05; 0.03) | 0.65 | -0.02 (-0.06; 0.03) | 0.47 | -0.01 (-0.04; 0.03) | 0.68 | -0.01 (-0.04; 0.03) | 0.69 | -0.01 (-0.04; 0.02) | 0.57 | -0.02 (-0.06; 0.01) | 0.16 |  |
| **Child non-verbal IQ** | 0.00 (-0.04; 0.03) | 0.82 | 0.00 (-0.04; 0.04) | 0.91 | 0.00 (-0.03; 0.04) | 0.86 | 0.01 (-0.03; 0.04) | 0.72 | 0.01 (-0.03; 0.04) | 0.65 | 0.00 (-0.04; 0.03) | 0.80 |  |
| **Child self-esteem** | -0.01 (-0.05; 0.03) | 0.54 | -0.01 (-0.05; 0.03) | 0.61 | 0.00 (-0.03; 0.04) | 0.96 | 0.02 (-0.01; 0.06) | 0.17 | 0.00 (-0.03; 0.04) | 0.88 | 0.00 (-0.03; 0.03) | 0.98 |  |
| **Maternal sensitivity** | - | - | - | - | - | - | - | - | - | - | - | - |  |
| **Friendship quality** | -0.02 (-0.06; 0.01) | 0.22 | 0.01 (-0.04; 0.05) | 0.8 | 0.02 (-0.02; 0.05) | 0.38 | 0.02 (-0.02; 0.05) | 0.38 | 0.01 (-0.02; 0.05) | 0.53 | 0.00 (-0.04; 0.03) | 0.91 |  |
| Note. Predictors included: cumulative adversity, protective factor (specific for each model), (child) sex, total intracranial volume, prenatal smoking, (child) age at the MRI scan, obstetric risk, and the interaction term between each protective factor and cumulative adversity. For the results of maternal sensitivity in Generation R see the main analyses. Negative affectivity scores in Generation R were reversed. | | | | | | | | | | | | | |
| All brain outcomes and adversity and protective factors were standardized. Abbreviations: ACC: Anterior cingulate cortex, OFC: Orbitofrontal cortex | | | | | | | | | | | | | |
| Generation R N=1,947 | | | | | | | | | | | | | |

| Table S10. Factor loadings in Generation R. | |  |  |  |
| --- | --- | --- | --- | --- |
|  |  | **3 component solution** | | |
|  |  | **RC1** | **RC3** | **RC2** |
| 1 | Early parenthood | **0.64** | -0.16 | 0.01 |
| 2 | One-parent family at child birth | **0.76** | 0.17 | 0.07 |
| 3 | Unwanted pregnancy | 0.28 | 0.07 | -0.06 |
| 4 | Parental psychopathology | 0.24 | **0.45** | 0.10 |
| 5 | Poverty | **0.67** | 0.18 | 0.08 |
| 6 | Parental death | 0.01 | 0.27 | -0.23 |
| 7 | Family relationship problems | -0.06 | **0.78** | -0.02 |
| 8 | Divorce/separation | **0.47** | **0.55** | 0.00 |
| 9 | Unemployment | 0.10 | **0.40** | 0.28 |
| 10 | Physical abuse to child | -0.03 | 0.05 | **0.63** |
| 11 | Psychological abuse | -0.10 | 0.23 | **0.65** |
| 12 | Sexual abuse | 0.11 | -0.12 | **0.57** |
|  | ***Variance explained per component*** | 0.15 | 0.13 | 0.11 |
| Note. Components are ordered based on the explained variance | | | |  |

| Table S11. Factor loadings in MARS. | |  |  |  |
| --- | --- | --- | --- | --- |
|  |  | **3 component solution** | | |
|  |  | **RC1** | **RC3** | **RC2** |
| 1 | Early parenthood | **0.69** | 0.17 | 0.12 |
| 2 | One-parent family at child birth | **0.80** | 0.10 | 0.05 |
| 3 | Unwanted pregnancy | **0.56** | 0.27 | 0.15 |
| 4 | Parental psychopathology | 0.15 | **0.75** | -0.01 |
| 5 | Poverty | **0.71** | 0.03 | -0.21 |
| 6 | Family relationship problems | -0.06 | **0.85** | 0.02 |
| 7 | Divorce/separation | 0.13 | 0.18 | 0.05 |
| 8 | Unemployment | 0.20 | **0.39** | -0.07 |
| 9 | Physical abuse to child | 0.08 | -0.07 | **0.83** |
| 10 | Psychological abuse | -0.01 | 0.06 | **0.78** |
|  | ***Variance explained per component*** | 0.20 | 0.16 | 0.14 |
| Note. Components are ordered based on the explained variance | | | |  |

| Table S12. Interaction between protective factors and **first** component of adversity in relation to brain outcomes (total: 3 components). | | | | | | | | | | |
| --- | --- | --- | --- | --- | --- | --- | --- | --- | --- | --- |
|  | *Global and regional brain outcomes* | | | | | | *Subcortical outcomes* | | | |
|  | Cortical grey matter volume | | Cerebral white matter volume | | Cerebellar volume | | Amygdala | | Hippocampus | |
|  | **β (95%CI)** | **p-value** | **β (95%CI)** | **p-value** | **β (95%CI)** | **p-value** | **β (95%CI)** | **p-value** | **β (95%CI)** | **p-value** |
| ***Generation R Study*** |  |  |  |  |  |  |  |  |  |  |
| **Temperament - Negative affectivity, reversed** | -0.01 (-0.05; 0.02) | 0.47 | -0.01 (-0.05; 0.03) | 0.67 | 0.01 (-0.02; 0.05) | 0.49 | -0.01 (-0.04; 0.03) | 0.62 | -0.02 (-0.06; 0.01) | 0.25 |
| **Temperament - Surgency** | 0.00 (-0.03; 0.04) | 0.84 | 0.00 (-0.03; 0.04) | 0.86 | 0.01 (-0.03; 0.05) | 0.73 | 0.01 (-0.02; 0.05) | 0.50 | -0.01 (-0.04; 0.03) | 0.67 |
| **Temperament - Effortful control** | 0.00 (-0.04; 0.03) | 0.83 | 0.00 (-0.04; 0.03) | 0.85 | -0.01 (-0.05; 0.03) | 0.60 | 0.00 (-0.04; 0.04) | 0.91 | 0.01 (-0.03; 0.04) | 0.62 |
| **Child non-verbal IQ** | -0.01 (-0.05; 0.03) | 0.50 | -0.01 (-0.05; 0.03) | 0.72 | -0.02 (-0.06; 0.02) | 0.36 | -0.01 (-0.04; 0.02) | 0.54 | 0.00 (-0.03; 0.03) | 0.99 |
| **Child self-esteem** | -0.01 (-0.05; 0.03) | 0.59 | 0.00 (-0.03; 0.04) | 0.97 | 0.00 (-0.03; 0.04) | 0.94 | 0.00 (-0.04; 0.03) | 0.94 | 0.00 (-0.03; 0.04) | 0.76 |
| **Maternal sensitivity*** | 0.01 (-0.09; 0.11) | 0.83 | 0.03 (-0.06; 0.13) | 0.49 | 0.00 (-0.10; 0.09) | 0.95 | 0.03 (-0.05; 0.11) | 0.46 | 0.02 (-0.07; 0.10) | 0.69 |
| **Friendship quality** | 0.02 (-0.02; 0.05) | 0.42 | 0.03 (-0.01; 0.07) | 0.10 | 0.03 (-0.01; 0.07) | 0.17 | 0.00 (-0.04; 0.04) | 0.97 | 0.00 (-0.03; 0.03) | 0.96 |
| ***MARS*** |  |  |  |  |  |  |  |  |  |  |
| **Temperament** |  |  |  |  |  |  |  |  |  |  |
| Temperament - easy/difficult trait | 0.00 (-0.12; 0.11) | 0.95 | 0.05 (-0.08; 0.17) | 0.45 | 0.04 (-0.08; 0.16) | 0.52 | 0.01 (-0.08; 0.11) | 0.82 | 0.01 (-0.09; 0.12) | 0.82 |
| Temperament - self-control | -0.02 (-0.12; 0.07) | 0.60 | 0.01 (-0.1; 0.11) | 0.92 | 0.05 (-0.05; 0.15) | 0.28 | -0.02 (-0.10; 0.06) | 0.65 | -0.03 (-0.12; 0.06) | 0.51 |
| **Child non-verbal IQ** | 0.00 (-0.10; 0.11) | 0.97 | 0.01 (-0.11; 0.12) | 0.89 | 0.02 (-0.1; 0.13) | 0.77 | -0.07 (-0.16; 0.02) | 0.13 | 0.05 (-0.05; 0.15) | 0.34 |
| **Child self-esteem** | 0.01 (-0.12; 0.13) | 0.94 | 0.06 (-0.08; 0.19) | 0.41 | 0.09 (-0.04; 0.23) | 0.17 | 0.07 (-0.04; 0.17) | 0.21 | 0.11 (0.00; 0.23) | 0.05 |
| **Maternal stimulation (sensitivity)**** | -0.01 (-0.11; 0.10) | 0.88 | 0.01 (-0.1; 0.12) | 0.80 | -0.01 (-0.12; 0.1) | 0.89 | 0.00 (-0.09; 0.08) | 0.93 | 0.01 (-0.09; 0.11) | 0.82 |
| Note. Predictors included: first adversity component, protective factor (specific for each model), sex, total intracranial volume (only in subcortical and prefrontal regions), prenatal smoking, maternal national origin (only in Generation R), age at the MRI scan (only in Generation R), obstetric risk, and the interaction term between each protective factor and cumulative adversity. Analyses with maternal sensitivity predictors in MARS additionally adjusted for child responsiveness. Analyses with maternal sensitivity in Generation R not adjusted for maternal national origin. Negative affectivity scores in Generation R were reversed. | | | | | | | | | | |
| All brain outcomes and adversity and protective factors were standardized. Amygdala and hippocampus volumes are the mean volumes across left and right hemisphere. Abbreviations: ACC: Anterior cingulate cortex, OFC: Orbitofrontal cortex. | | | | | | | | | | |
| Generation R N = 3,008. *Analyses with maternal sensitivity performed in N = 383. | | | | | | | | | | |
| MARS N = 179. **Analyses with maternal sensitivity performed in N = 173. | | | | | | | | | | |

| Table S12. Interaction between protective factors and **first** component of adversity in relation to brain outcomes (total: 3 components) (*cont.*). | | | | | | | | | | | | |
| --- | --- | --- | --- | --- | --- | --- | --- | --- | --- | --- | --- | --- |
|  | Left ACC | | Right ACC | | Left medial OFC | | Right medial OFC | | Left rostral middle frontal cortex | | Right rostral middle frontal cortex | |
|  | **β (95%CI)** | **p-value** | **β (95%CI)** | **p-value** | **β (95%CI)** | **p-value** | **β (95%CI)** | **p-value** | **β (95%CI)** | **p-value** | **β (95%CI)** | **p-value** |
| ***Generation R Study*** |  |  |  |  |  |  |  |  |  |  |  |  |
| **Temperament - Negative affectivity, reversed** | 0.00 (-0.03; 0.04) | 0.83 | -0.02 (-0.05; 0.02) | 0.38 | 0.00 (-0.03; 0.04) | 0.86 | -0.01 (-0.04; 0.02) | 0.53 | -0.02 (-0.05; 0.02) | 0.31 | -0.01 (-0.05; 0.02) | 0.38 |
| **Temperament - Surgency** | -0.01 (-0.05; 0.02) | 0.49 | 0.00 (-0.04; 0.04) | 0.94 | 0.01 (-0.03; 0.04) | 0.77 | 0.01 (-0.02; 0.04) | 0.53 | 0.01 (-0.02; 0.04) | 0.54 | 0.01 (-0.02; 0.05) | 0.36 |
| **Temperament - Effortful control** | 0.01 (-0.03; 0.04) | 0.79 | 0.00 (-0.03; 0.04) | 0.87 | -0.01 (-0.04; 0.03) | 0.64 | 0.00 (-0.04; 0.03) | 0.77 | 0.00 (-0.03; 0.04) | 0.88 | 0.00 (-0.03; 0.03) | 0.98 |
| **Child non-verbal IQ** | 0.01 (-0.03; 0.05) | 0.54 | 0.01 (-0.03; 0.05) | 0.58 | -0.01 (-0.04; 0.03) | 0.68 | -0.01 (-0.04; 0.03) | 0.75 | -0.01 (-0.04; 0.02) | 0.52 | 0.00 (-0.04; 0.03) | 0.78 |
| **Child self-esteem** | 0.00 (-0.03; 0.03) | 0.98 | 0.00 (-0.03; 0.04) | 0.99 | 0.00 (-0.03; 0.03) | 0.94 | -0.01 (-0.04; 0.03) | 0.74 | -0.01 (-0.03; 0.02) | 0.62 | -0.01 (-0.04; 0.02) | 0.44 |
| **Maternal sensitivity*** | -0.03 (-0.12; 0.07) | 0.56 | 0.04 (-0.06; 0.15) | 0.40 | 0.04 (-0.05; 0.13) | 0.38 | 0.06 (-0.03; 0.14) | 0.20 | 0.00 (-0.08; 0.09) | 0.97 | -0.03 (-0.11; 0.06) | 0.53 |
| **Friendship quality** | -0.01 (-0.05; 0.03) | 0.59 | 0.00 (-0.04; 0.04) | 0.83 | -0.02 (-0.06; 0.02) | 0.29 | 0.01 (-0.03; 0.04) | 0.66 | -0.02 (-0.06; 0.02) | 0.36 | -0.02 (-0.06; 0.01) | 0.26 |
| ***MARS*** |  |  |  |  |  |  |  |  |  |  |  |  |
| **Temperament** |  |  |  |  |  |  |  |  |  |  |  |  |
| Temperament - easy/difficult trait | 0.00 (-0.12; 0.12) | 0.98 | 0.03 (-0.09; 0.16) | 0.61 | -0.12 (-0.23; -0.02) | 0.02 | -0.04 (-0.14; 0.07) | 0.51 | 0.00 (-0.10; 0.10) | 1.00 | -0.06 (-0.15; 0.03) | 0.19 |
| Temperament - self-control | 0.05 (-0.04; 0.15) | 0.28 | 0.04 (-0.07; 0.14) | 0.47 | -0.02 (-0.11; 0.06) | 0.59 | 0.01 (-0.07; 0.1) | 0.75 | -0.01 (-0.1; 0.07) | 0.73 | 0.00 (-0.07; 0.08) | 0.96 |
| **Child non-verbal IQ** | -0.02 (-0.13; 0.10) | 0.78 | 0.03 (-0.09; 0.14) | 0.67 | 0.01 (-0.09; 0.11) | 0.78 | 0.01 (-0.09; 0.11) | 0.80 | -0.02 (-0.11; 0.08) | 0.75 | -0.06 (-0.15; 0.02) | 0.15 |
| **Child self-esteem** | 0.03 (-0.10; 0.16) | 0.66 | 0.03 (-0.11; 0.18) | 0.63 | 0.02 (-0.10; 0.13) | 0.79 | 0.00 (-0.12; 0.12) | 0.99 | 0.02 (-0.09; 0.13) | 0.71 | -0.03 (-0.13; 0.08) | 0.62 |
| **Maternal stimulation (sensitivity)**** | 0.01 (-0.10; 0.12) | 0.88 | 0.00 (-0.12; 0.11) | 0.93 | -0.03 (-0.13; 0.07) | 0.53 | 0.01 (-0.08; 0.11) | 0.79 | 0.02 (-0.07; 0.11) | 0.73 | 0.00 (-0.08; 0.08) | 0.97 |
| Note. Predictors included: first adversity component, protective factor (specific for each model), sex, total intracranial volume (only in subcortical and prefrontal regions), prenatal smoking, maternal national origin (only in Generation R), age at the MRI scan (only in Generation R), obstetric risk, and the interaction term between each protective factor and cumulative adversity. Analyses with maternal sensitivity predictors in MARS additionally adjusted for child responsiveness. Analyses with maternal sensitivity in Generation R not adjusted for maternal national origin. Negative affectivity scores in Generation R were reversed. | | | | | | | | | | | | |
| All brain outcomes and adversity and protective factors were standardized. Amygdala and hippocampus volumes are the mean volumes across left and right hemisphere. Abbreviations: ACC: Anterior cingulate cortex, OFC: Orbitofrontal cortex. | | | | | | | | | | | | |
| Generation R N = 3,008. *Analyses with maternal sensitivity performed in N = 383. | | | | | | | | | | | | |
| MARS N = 179. **Analyses with maternal sensitivity performed in N = 173. | | | | | | | | | | | | |

| Table S13. Interaction between protective factors and **second** component of adversity in relation to brain outcomes (total: 3 components). | | | | | | | | | | |
| --- | --- | --- | --- | --- | --- | --- | --- | --- | --- | --- |
|  | *Global and regional brain outcomes* | | | | | | *Subcortical outcomes* | | | |
|  | Cortical grey matter volume | | Cerebral white matter volume | | Cerebellar volume | | Amygdala | | Hippocampus | |
|  | **β (95%CI)** | **p-value** | **β (95%CI)** | **p-value** | **β (95%CI)** | **p-value** | **β (95%CI)** | **p-value** | **β (95%CI)** | **p-value** |
| ***Generation R Study*** |  |  |  |  |  |  |  |  |  |  |
| **Temperament - Negative affectivity, reversed** | -0.03 (-0.07; 0.01) | 0.13 | -0.01 (-0.04; 0.03) | 0.70 | -0.01 (-0.05; 0.02) | 0.47 | 0.00 (-0.03; 0.03) | 0.95 | -0.01 (-0.04; 0.03) | 0.73 |
| **Temperament - Surgency** | 0.01 (-0.03; 0.05) | 0.59 | 0.01 (-0.03; 0.05) | 0.64 | 0.02 (-0.02; 0.06) | 0.32 | 0.00 (-0.03; 0.03) | 0.93 | 0.00 (-0.03; 0.03) | 0.87 |
| **Temperament - Effortful control** | 0.00 (-0.04; 0.03) | 0.81 | 0.00 (-0.03; 0.04) | 0.86 | 0.00 (-0.03; 0.04) | 0.82 | 0.00 (-0.03; 0.03) | 0.89 | 0.01 (-0.02; 0.04) | 0.61 |
| **Child non-verbal IQ** | -0.01 (-0.04; 0.02) | 0.49 | -0.01 (-0.04; 0.03) | 0.71 | -0.01 (-0.04; 0.02) | 0.45 | -0.01 (-0.04; 0.02) | 0.56 | -0.02 (-0.05; 0.01) | 0.15 |
| **Child self-esteem** | 0.00 (-0.03; 0.03) | 0.97 | 0.01 (-0.03; 0.04) | 0.71 | -0.01 (-0.05; 0.02) | 0.40 | -0.02 (-0.05; 0.01) | 0.24 | -0.01 (-0.03; 0.02) | 0.69 |
| **Maternal sensitivity*** | -0.04 (-0.12; 0.04) | 0.37 | -0.08 (-0.16; 0.00) | 0.05 | -0.05 (-0.13; 0.03) | 0.19 | 0.01 (-0.06; 0.08) | 0.81 | -0.04 (-0.11; 0.03) | 0.29 |
| **Friendship quality** | 0.01 (-0.02; 0.04) | 0.60 | 0.01 (-0.02; 0.04) | 0.58 | 0.00 (-0.03; 0.04) | 0.87 | -0.02 (-0.05; 0.01) | 0.29 | -0.01 (-0.04; 0.02) | 0.51 |
| ***MARS*** |  |  |  |  |  |  |  |  |  |  |
| **Temperament** |  |  |  |  |  |  |  |  |  |  |
| Temperament - easy/difficult trait | 0.01 (-0.12; 0.15) | 0.86 | 0.01 (-0.14; 0.16) | 0.86 | -0.03 (-0.17; 0.12) | 0.70 | -0.02 (-0.13; 0.10) | 0.76 | -0.05 (-0.17; 0.08) | 0.46 |
| Temperament - self-control | -0.01 (-0.14; 0.13) | 0.92 | -0.01 (-0.16; 0.13) | 0.84 | -0.06 (-0.20; 0.09) | 0.44 | 0.00 (-0.11; 0.11) | 1.00 | 0.03 (-0.10; 0.15) | 0.65 |
| **Child non-verbal IQ** | -0.03 (-0.16; 0.10) | 0.61 | -0.02 (-0.16; 0.12) | 0.81 | -0.03 (-0.17; 0.11) | 0.65 | -0.03 (-0.14; 0.08) | 0.63 | -0.15 (-0.27; -0.03) | 0.02 |
| **Child self-esteem** | -0.06 (-0.19; 0.07) | 0.37 | 0.01 (-0.14; 0.15) | 0.91 | 0.04 (-0.10; 0.18) | 0.60 | 0.00 (-0.11; 0.11) | 0.97 | -0.01 (-0.13; 0.12) | 0.90 |
| **Maternal stimulation (sensitivity)**** | -0.04 (-0.15; 0.08) | 0.54 | 0.00 (-0.13; 0.12) | 0.95 | 0.06 (-0.06; 0.18) | 0.33 | 0.00 (-0.10; 0.09) | 0.92 | -0.02 (-0.13; 0.08) | 0.70 |
| Note. Predictors included: second adversity component, protective factor (specific for each model), sex, total intracranial volume (only in subcortical and prefrontal regions), prenatal smoking, maternal national origin (only in Generation R), age at the MRI scan (only in Generation R), obstetric risk, and the interaction term between each protective factor and cumulative adversity. Analyses with maternal sensitivity predictors in MARS additionally adjusted for child responsiveness. Analyses with maternal sensitivity in Generation R not adjusted for maternal national origin. Negative affectivity scores in Generation R were reversed. | | | | | | | | | | |
| All brain outcomes and adversity and protective factors were standardized. Amygdala and hippocampus volumes are the mean volumes across left and right hemisphere. Abbreviations: ACC: Anterior cingulate cortex, OFC: Orbitofrontal cortex. | | | | | | | | | | |
| Generation R N = 3,008. *Analyses with maternal sensitivity performed in N = 383. | | | | | | | | | | |
| MARS N = 179. **Analyses with maternal sensitivity performed in N = 173. | | | | | | | | | | |

| Table S13. Interaction between protective factors and **second** component of adversity in relation to brain outcomes (total: 3 components) *(cont.).* | | | | | | | | | | | | |
| --- | --- | --- | --- | --- | --- | --- | --- | --- | --- | --- | --- | --- |
|  | Left ACC | | Right ACC | | Left medial OFC | | Right medial OFC | | Left rostral middle frontal cortex | | Right rostral middle frontal cortex | |
|  | **β (95%CI)** | **p-value** | **β (95%CI)** | **p-value** | **β (95%CI)** | **p-value** | **β (95%CI)** | **p-value** | **β (95%CI)** | **p-value** | **β (95%CI)** | **p-value** |
| ***Generation R Study*** |  |  |  |  |  |  |  |  |  |  |  |  |
| **Temperament - Negative affectivity, reversed** | 0.01 (-0.03; 0.04) | 0.73 | 0.01 (-0.03; 0.05) | 0.68 | -0.02 (-0.06; 0.01) | 0.22 | 0.00 (-0.04; 0.03) | 0.90 | -0.02 (-0.05; 0.01) | 0.25 | -0.02 (-0.05; 0.02) | 0.36 |
| **Temperament - Surgency** | -0.01 (-0.04; 0.03) | 0.73 | 0.02 (-0.02; 0.06) | 0.42 | 0.00 (-0.04; 0.03) | 0.80 | -0.01 (-0.04; 0.02) | 0.44 | 0.01 (-0.02; 0.04) | 0.50 | 0.01 (-0.02; 0.04) | 0.35 |
| **Temperament - Effortful control** | 0.00 (-0.04; 0.03) | 0.78 | 0.00 (-0.04; 0.04) | 0.98 | -0.01 (-0.04; 0.03) | 0.64 | -0.01 (-0.04; 0.02) | 0.60 | 0.02 (-0.01; 0.05) | 0.30 | -0.02 (-0.05; 0.01) | 0.24 |
| **Child non-verbal IQ** | -0.03 (-0.06; 0.00) | 0.07 | -0.01 (-0.05; 0.02) | 0.55 | 0.00 (-0.04; 0.03) | 0.76 | 0.00 (-0.03; 0.03) | 0.99 | 0.00 (-0.03; 0.03) | 0.99 | -0.02 (-0.05; 0.01) | 0.20 |
| **Child self-esteem** | -0.02 (-0.05; 0.01) | 0.24 | -0.01 (-0.05; 0.02) | 0.51 | -0.02 (-0.05; 0.01) | 0.23 | 0.00 (-0.03; 0.03) | 0.79 | 0.01 (-0.02; 0.04) | 0.55 | 0.00 (-0.03; 0.03) | 0.94 |
| **Maternal sensitivity*** | 0.03 (-0.04; 0.11) | 0.41 | 0.06 (-0.02; 0.14) | 0.16 | 0.08 (0.01; 0.15) | 0.03 | 0.06 (-0.01; 0.14) | 0.10 | 0.02 (-0.04; 0.09) | 0.53 | 0.04 (-0.02; 0.11) | 0.21 |
| **Friendship quality** | 0.00 (-0.04; 0.03) | 0.78 | 0.02 (-0.01; 0.06) | 0.19 | 0.00 (-0.03; 0.04) | 0.78 | -0.01 (-0.04; 0.02) | 0.35 | 0.00 (-0.02; 0.03) | 0.77 | 0.00 (-0.03; 0.03) | 0.89 |
| ***MARS*** |  |  |  |  |  |  |  |  |  |  |  |  |
| **Temperament** |  |  |  |  |  |  |  |  |  |  |  |  |
| Temperament - easy/difficult trait | 0.07 (-0.07; 0.21) | 0.31 | -0.07 (-0.21; 0.07) | 0.34 | -0.01 (-0.14; 0.11) | 0.84 | -0.05 (-0.17; 0.08) | 0.46 | 0.03 (-0.09; 0.15) | 0.58 | 0.06 (-0.05; 0.17) | 0.27 |
| Temperament - self-control | -0.04 (-0.18; 0.10) | 0.57 | 0.02 (-0.12; 0.16) | 0.82 | -0.02 (-0.14; 0.10) | 0.75 | -0.13 (-0.25; -0.01) | 0.04 | -0.09 (-0.20; 0.03) | 0.15 | -0.05 (-0.16; 0.06) | 0.34 |
| **Child non-verbal IQ** | -0.08 (-0.21; 0.06) | 0.28 | -0.04 (-0.18; 0.10) | 0.53 | -0.04 (-0.16; 0.09) | 0.57 | -0.05 (-0.18; 0.07) | 0.39 | 0.03 (-0.09; 0.15) | 0.62 | 0.03 (-0.08; 0.13) | 0.62 |
| **Child self-esteem** | -0.04 (-0.18; 0.10) | 0.56 | -0.01 (-0.15; 0.14) | 0.91 | 0.06 (-0.07; 0.19) | 0.34 | 0.01 (-0.12; 0.13) | 0.90 | -0.01 (-0.13; 0.11) | 0.88 | -0.04 (-0.15; 0.07) | 0.45 |
| **Maternal stimulation (sensitivity)**** | -0.05 (-0.17; 0.07) | 0.42 | 0.01 (-0.11; 0.13) | 0.85 | 0.01 (-0.10; 0.11) | 0.92 | -0.08 (-0.18; 0.03) | 0.15 | 0.01 (-0.09; 0.11) | 0.86 | 0.00 (-0.09; 0.09) | 0.96 |
| Note. Predictors included: second adversity component, protective factor (specific for each model), sex, total intracranial volume (only in subcortical and prefrontal regions), prenatal smoking, maternal national origin (only in Generation R), age at the MRI scan (only in Generation R), obstetric risk, and the interaction term between each protective factor and cumulative adversity. Analyses with maternal sensitivity predictors in MARS additionally adjusted for child responsiveness. Analyses with maternal sensitivity in Generation R not adjusted for maternal national origin. Negative affectivity scores in Generation R were reversed. | | | | | | | | | | | | |
| All brain outcomes and adversity and protective factors were standardized. Amygdala and hippocampus volumes are the mean volumes across left and right hemisphere. Abbreviations: ACC: Anterior cingulate cortex, OFC: Orbitofrontal cortex. | | | | | | | | | | | | |
| Generation R N = 3,008. *Analyses with maternal sensitivity performed in N = 383. | | | | | | | | | | | | |
| MARS N = 179. **Analyses with maternal sensitivity performed in N = 173. | | | | | | | | | | | | |

| Table S14. Interaction between protective factors and **third** component of adversity in relation to brain outcomes (total: 3 components). | | | | | | | | | | |
| --- | --- | --- | --- | --- | --- | --- | --- | --- | --- | --- |
|  | *Global and regional brain outcomes* | | | | | | *Subcortical outcomes* | | | |
|  | Cortical grey matter volume | | Cerebral white matter volume | | Cerebellar volume | | Amygdala | | Hippocampus | |
|  | **β (95%CI)** | **p-value** | **β (95%CI)** | **p-value** | **β (95%CI)** | **p-value** | **β (95%CI)** | **p-value** | **β (95%CI)** | **p-value** |
| ***Generation R Study*** |  |  |  |  |  |  |  |  |  |  |
| **Temperament - Negative affectivity, reversed** | 0.00 (-0.03; 0.04) | 0.87 | 0.01 (-0.03; 0.04) | 0.70 | 0.01 (-0.03; 0.04) | 0.69 | 0.00 (-0.03; 0.03) | 0.96 | 0.00 (-0.03; 0.03) | 0.96 |
| **Temperament - Surgency** | 0.01 (-0.03; 0.05) | 0.62 | -0.01 (-0.05; 0.03) | 0.62 | 0.00 (-0.03; 0.04) | 0.82 | 0.01 (-0.03; 0.04) | 0.74 | 0.00 (-0.04; 0.03) | 0.78 |
| **Temperament - Effortful control** | -0.02 (-0.05; 0.02) | 0.32 | -0.01 (-0.04; 0.03) | 0.65 | 0.02 (-0.03; 0.06) | 0.46 | 0.01 (-0.03; 0.04) | 0.72 | 0.02 (-0.01; 0.05) | 0.26 |
| **Child non-verbal IQ** | -0.01 (-0.05; 0.02) | 0.41 | -0.02 (-0.05; 0.02) | 0.38 | 0.01 (-0.03; 0.05) | 0.71 | -0.01 (-0.04; 0.02) | 0.58 | -0.01 (-0.04; 0.02) | 0.56 |
| **Child self-esteem** | 0.02 (-0.01; 0.06) | 0.22 | 0.02 (-0.02; 0.05) | 0.33 | 0.02 (-0.02; 0.05) | 0.34 | 0.01 (-0.02; 0.04) | 0.46 | 0.01 (-0.02; 0.04) | 0.56 |
| **Maternal sensitivity*** | 0.07 (-0.02; 0.17) | 0.13 | 0.04 (-0.05; 0.13) | 0.39 | 0.02 (-0.07; 0.12) | 0.63 | 0.05 (-0.03; 0.13) | 0.21 | 0.03 (-0.05; 0.11) | 0.46 |
| **Friendship quality** | 0.02 (-0.02; 0.05) | 0.37 | 0.02 (-0.02; 0.05) | 0.32 | 0.03 (0.00; 0.07) | 0.08 | -0.02 (-0.05; 0.02) | 0.29 | -0.01 (-0.04; 0.02) | 0.59 |
| ***MARS*** |  |  |  |  |  |  |  |  |  |  |
| **Temperament** |  |  |  |  |  |  |  |  |  |  |
| Temperament - easy/difficult trait | 0.01 (-0.10; 0.13) | 0.83 | -0.01 (-0.13; 0.12) | 0.90 | -0.01 (-0.13; 0.11) | 0.83 | -0.06 (-0.16; 0.03) | 0.20 | -0.06 (-0.16; 0.05) | 0.30 |
| Temperament - self-control | 0.04 (-0.09; 0.16) | 0.56 | 0.02 (-0.12; 0.15) | 0.79 | 0.04 (-0.09; 0.17) | 0.52 | 0.00 (-0.11; 0.10) | 0.97 | -0.01 (-0.13; 0.10) | 0.85 |
| **Child non-verbal IQ** | 0.02 (-0.10; 0.14) | 0.73 | 0.05 (-0.08; 0.17) | 0.46 | -0.07 (-0.19; 0.05) | 0.28 | -0.08 (-0.18; 0.02) | 0.12 | -0.06 (-0.17; 0.05) | 0.32 |
| **Child self-esteem** | 0.00 (-0.12; 0.11) | 0.96 | 0.00 (-0.13; 0.13) | 0.98 | 0.02 (-0.11; 0.14) | 0.78 | 0.03 (-0.07; 0.13) | 0.56 | -0.02 (-0.13; 0.09) | 0.67 |
| **Maternal stimulation (sensitivity)**** | 0.04 (-0.07; 0.14) | 0.49 | 0.07 (-0.04; 0.19) | 0.19 | -0.01 (-0.12; 0.10) | 0.81 | 0.02 (-0.07; 0.11) | 0.62 | 0.05 (-0.05; 0.14) | 0.37 |
| Note. Predictors included: third adversity component, protective factor (specific for each model), sex, total intracranial volume (only in subcortical and prefrontal regions), prenatal smoking, maternal national origin (only in Generation R), age at the MRI scan (only in Generation R), obstetric risk, and the interaction term between each protective factor and cumulative adversity. Analyses with maternal sensitivity predictors in MARS additionally adjusted for child responsiveness. Analyses with maternal sensitivity in Generation R not adjusted for maternal national origin. Negative affectivity scores in Generation R were reversed. | | | | | | | | | | |
| All brain outcomes and adversity and protective factors were standardized. Amygdala and hippocampus volumes are the mean volumes across left and right hemisphere. Abbreviations: ACC: Anterior cingulate cortex, OFC: Orbitofrontal cortex. | | | | | | | | | | |
| Generation R N = 3,008. *Analyses with maternal sensitivity performed in N = 383. | | | | | | | | | | |
| MARS N = 179. **Analyses with maternal sensitivity performed in N = 173. | | | | | | | | | | |

| Table S14. Interaction between protective factors and **third** component of adversity in relation to brain outcomes (total: 3 components) *(cont).* | | | | | | | | | | | | |
| --- | --- | --- | --- | --- | --- | --- | --- | --- | --- | --- | --- | --- |
|  | Left ACC | | Right ACC | | Left medial OFC | | Right medial OFC | | Left rostral middle frontal cortex | | Right rostral middle frontal cortex | |
|  | **β (95%CI)** | **p-value** | **β (95%CI)** | **p-value** | **β (95%CI)** | **p-value** | **β (95%CI)** | **p-value** | **β (95%CI)** | **p-value** | **β (95%CI)** | **p-value** |
| ***Generation R Study*** |  |  |  |  |  |  |  |  |  |  |  |  |
| **Temperament - Negative affectivity, reversed** | 0.00 (-0.03; 0.04) | 0.97 | -0.02 (-0.06; 0.01) | 0.26 | -0.02 (-0.06; 0.01) | 0.23 | -0.01 (-0.05; 0.02) | 0.39 | 0.01 (-0.02; 0.04) | 0.55 | 0.00 (-0.03; 0.04) | 0.93 |
| **Temperament - Surgency** | 0.01 (-0.02; 0.05) | 0.41 | 0.00 (-0.04; 0.04) | 0.99 | 0.00 (-0.04; 0.03) | 0.97 | 0.00 (-0.03; 0.04) | 0.94 | 0.00 (-0.04; 0.03) | 0.84 | 0.00 (-0.03; 0.04) | 0.89 |
| **Temperament - Effortful control** | -0.02 (-0.06; 0.01) | 0.21 | -0.01 (-0.05; 0.02) | 0.46 | -0.01 (-0.04; 0.03) | 0.68 | 0.00 (-0.03; 0.03) | 0.82 | -0.02 (-0.05; 0.01) | 0.29 | -0.02 (-0.05; 0.01) | 0.25 |
| **Child non-verbal IQ** | -0.02 (-0.05; 0.02) | 0.37 | -0.01 (-0.05; 0.02) | 0.52 | -0.01 (-0.04; 0.03) | 0.66 | -0.01 (-0.04; 0.02) | 0.41 | 0.01 (-0.02; 0.04) | 0.70 | -0.01 (-0.05; 0.02) | 0.35 |
| **Child self-esteem** | 0.00 (-0.04; 0.03) | 0.90 | 0.01 (-0.03; 0.04) | 0.60 | 0.01 (-0.02; 0.05) | 0.44 | 0.03 (-0.01; 0.06) | 0.15 | 0.00 (-0.02; 0.03) | 0.78 | 0.00 (-0.03; 0.03) | 0.85 |
| **Maternal sensitivity*** | -0.01 (-0.1; 0.09) | 0.90 | 0.00 (-0.1; 0.1) | 0.95 | -0.01 (-0.10; 0.07) | 0.80 | 0.04 (-0.04; 0.12) | 0.36 | 0.07 (-0.01; 0.15) | 0.10 | 0.01 (-0.07; 0.09) | 0.81 |
| **Friendship quality** | -0.03 (-0.06; 0.01) | 0.14 | 0.01 (-0.03; 0.04) | 0.74 | 0.01 (-0.02; 0.05) | 0.48 | 0.02 (-0.01; 0.05) | 0.28 | 0.02 (-0.01; 0.05) | 0.25 | 0.01 (-0.02; 0.04) | 0.63 |
| ***MARS*** |  |  |  |  |  |  |  |  |  |  |  |  |
| **Temperament** |  |  |  |  |  |  |  |  |  |  |  |  |
| Temperament - easy/difficult trait | 0.03 (-0.09; 0.15) | 0.65 | -0.07 (-0.19; 0.06) | 0.30 | -0.05 (-0.16; 0.05) | 0.34 | -0.07 (-0.17; 0.04) | 0.22 | -0.05 (-0.15; 0.05) | 0.30 | 0.01 (-0.08; 0.11) | 0.78 |
| Temperament - self-control | -0.07 (-0.2; 0.06) | 0.28 | 0.03 (-0.11; 0.16) | 0.70 | -0.05 (-0.16; 0.07) | 0.43 | -0.05 (-0.16; 0.07) | 0.43 | 0.02 (-0.09; 0.12) | 0.78 | 0.00 (-0.10; 0.10) | 0.93 |
| **Child non-verbal IQ** | -0.04 (-0.16; 0.08) | 0.52 | 0.09 (-0.04; 0.22) | 0.18 | 0.03 (-0.08; 0.14) | 0.62 | -0.04 (-0.15; 0.07) | 0.47 | -0.04 (-0.14; 0.06) | 0.47 | -0.09 (-0.19; 0.00) | 0.06 |
| **Child self-esteem** | -0.01 (-0.13; 0.12) | 0.91 | 0.10 (-0.03; 0.23) | 0.12 | -0.01 (-0.13; 0.1) | 0.80 | -0.08 (-0.19; 0.02) | 0.13 | 0.06 (-0.04; 0.16) | 0.27 | 0.04 (-0.05; 0.14) | 0.37 |
| **Maternal stimulation (sensitivity)**** | -0.02 (-0.13; 0.09) | 0.78 | -0.01 (-0.13; 0.1) | 0.81 | -0.02 (-0.12; 0.08) | 0.63 | 0.08 (-0.01; 0.18) | 0.10 | -0.01 (-0.1; 0.08) | 0.85 | 0.04 (-0.04; 0.13) | 0.30 |
| Note. Predictors included: third adversity component, protective factor (specific for each model), sex, total intracranial volume (only in subcortical and prefrontal regions), prenatal smoking, maternal national origin (only in Generation R), age at the MRI scan (only in Generation R), obstetric risk, and the interaction term between each protective factor and cumulative adversity. Analyses with maternal sensitivity predictors in MARS additionally adjusted for child responsiveness. Analyses with maternal sensitivity in Generation R not adjusted for maternal national origin. Negative affectivity scores in Generation R were reversed. | | | | | | | | | | | | |
| All brain outcomes and adversity and protective factors were standardized. Amygdala and hippocampus volumes are the mean volumes across left and right hemisphere. Abbreviations: ACC: Anterior cingulate cortex, OFC: Orbitofrontal cortex. | | | | | | | | | | | | |
| Generation R N = 3,008. *Analyses with maternal sensitivity performed in N = 383. | | | | | | | | | | | | |
| MARS N = 179. **Analyses with maternal sensitivity performed in N = 173. | | | | | | | | | | | | |
